# Computational discovery of regulatory elements in a continuous expression space

**DOI:** 10.1186/gb-2012-13-11-r109

**Published:** 2012-11-27

**Authors:** Mathieu Lajoie, Olivier Gascuel, Vincent Lefort, Laurent Bréhélin

**Affiliations:** 1 Méthodes et algorithmes pour la Bioinformatique, LIRMM, Univ. Montpellier 2, CNRS; 161 rue Ada, 34095 MONTPELLIER, France

**Keywords:** motif discovery, gene regulation, co-expressed genes, clustering, k-nearest neighbors

## Abstract

Approaches for regulatory element discovery from gene expression data usually rely on clustering algorithms to partition the data into clusters of co-expressed genes. Gene regulatory sequences are then mined to find overrepresented motifs in each cluster. However, this ad hoc partition rarely fits the biological reality. We propose a novel method called RED^2 ^that avoids data clustering by estimating motif densities locally around each gene. We show that RED^2 ^detects numerous motifs not detected by clustering-based approaches, and that most of these correspond to characterized motifs. RED^2 ^can be accessed online through a user-friendly interface.

## Background

Gene expression is modulated depending on time, space and environmental conditions. This process involves many levels, including chromatin structure, transcription, transcript stability or localization, and translation. At most of these levels, regulation is achieved through the binding of regulatory proteins with specific short nucleic acid sequences called regulatory elements (REs). A notable exception is non-coding RNA mediated regulation [1].

Discovering REs (that is, their associated sequence motifs) from gene expression and sequence data can be done in several ways. One of the most common approaches is to use a clustering algorithm to partition the expression dataset, and to apply, on each cluster, one of the numerous algorithms that have been designed to find overrepresented motifs in a predefined set of sequences, such as MEME [2], AlignACE [3] or Weeder [4] (see Sandve *et al. *[5] for a comparison of their performance). There are two major limitations to this approach.

First, most of these algorithms rely on statistical models of sequence background, which have been reported to produce many false positives [6,7], especially with repeat-rich and atypical genomes. For example, this is the case with *Plasmodium falciparum *- the main causal agent of human malaria - whose A+T content reaches almost 90% in intergenic regions [8].

**Figure 1:**
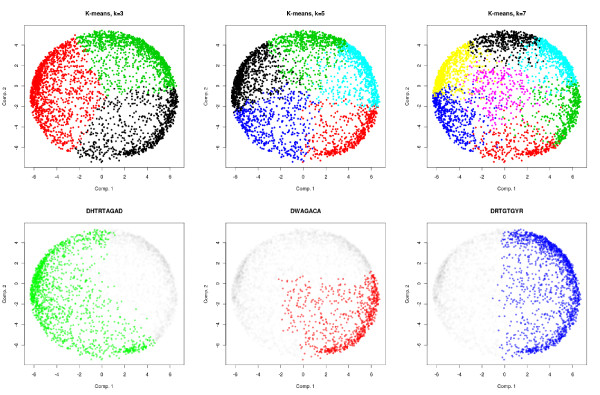
**Gene expression data of *P. falciparum *48 h erythrocytic cycle **[11]. Each point in the figures corresponds to an expression profile plotted according to the first and second principal components. All points remain at the same coordinate throughout the six figures (that is, only the coloring changes). In the top row, colors indicate the cluster membership of each profile after k-means clustering of the original data (that is, prior to dimensionality reduction) using a Pearson correlation distance for *k *= 3, 5 and 7. We observe that the clusters are nearly equally sized and that their edges are rather arbitrary, as they do not follow low density regions and change radically for different *k *values. Figures in the bottom row show regions of the expression space enriched for three regulatory motifs identified in previous studies [6,7,18]. Enrichment is defined on the original data (that is, prior to dimensionality reduction) by measuring the proportion of genes that contain the motif in their upstream sequence (1 kb) among the 200 nearest neighbors of each gene (according to their profiles). Colored points correspond to profiles where this proportion is three standard deviations above the expected one according to the hypergeometric law (see motif density in Material and methods). Uncolored points are shaded for clarity. We observe that: (i) each motif corresponds to a contiguous region of the expression space, (ii) these regions do not correspond to those defined by any of the above clusterings and (iii) regions defined by different motifs can strongly overlap, highlighting the weaknesses of clustering-based approaches for motif discovery.

Second, clustering assumes that: (i) a natural partition of the genes in well-defined clusters exists and can be inferred from the data, and (ii) co-regulated gene sets are disjoint. In fact, determining the real number of clusters in a dataset is considered to be one of the hardest classification problems, and has been the topic of numerous studies. Moreover, it is a well-known fact that different regulatory motifs can have overlapping gene sets [9,10]. These two points are illustrated in Figure 1 with the *P. falciparum *intraerythrocytic gene expression data of Bozdech *et al. *[11]. The periodic nature of this expression dataset allows projection on a two-dimensional space with minimal loss of variance using principal component analysis. The upper part of the figure represents the partitions obtained after performing k-means clustering of the genes in the original space using different numbers of clusters. We see that the clusters have similar shapes and sizes and that their edges arbitrarily change depending on the number of clusters. The resulting beach-ball-like partitions of the expression space suggest that there is no natural clustering in this dataset. In the bottom part of the figure, we show the regions of the expression space enriched for three regulatory motifs identified in previous studies [6,7]. We see that the regions defined for the different motifs clearly overlap and do not correspond to the regions defined by any of the above clusterings.

Several methods that do not require a clustering have also been developed. For example, RankMotif++ [12] and DRIM [13] detect motif imbalance in ranked lists of genes obtained by ordering expression changes between two conditions. These methods have the advantage of not relying on a statistical model of background sequence, as they consider the motif distribution in the whole set of available sequences. However, although well suited for two-condition datasets, a single ordering can hardly reflect all the information contained in high-dimensional datasets, for example, those obtained by measuring expression in multiple conditions or at different time points, as routinely produced nowadays. Similarly, methods like REDUCE [14] and MatrixREDUCE [15] do not rely on clustering. They use linear regression to identify motifs that can be used to predict expression levels, assuming that the latter are linearly correlated with the number of motif occurrences in each sequence. However, as noted in Elemento *et al. *[6], the validity of this assumption has not been widely explored, nor the behavior of such approaches in case of violation. Finally, we mention the early work of Holmes and Bruno [16], which discovers the clustering and the motifs simultaneously. Although this approach seems very appealing, it has not been tested on real datasets, probably because of its prohibitive running time [16].

The FIRE [6] and GEMS [7] algorithms have been specifically designed for finding REs from whole genomes and high-dimensional datasets. They differ advantageously from DRIM and RankMotif++ as they are not restricted to ranked lists of genes, and from REDUCE and MatrixREDUCE as they make as few assumptions as possible about the way in which REs drive expression. Compared to MEME and AlignACE, they do not use a background model and are less prone to reporting false positives. However, they both rely on expression-data clustering, and thus are subject to the criticisms mentioned above. GEMS differs from FIRE in the way the dependency between the presence of a motif and the expression profile of the corresponding gene is measured. Specifically, GEMS uses the hypergeometric distribution to assess motif enrichment in each cluster, while FIRE computes the mutual information between the presence/absence of a motif and the cluster membership of the corresponding sequences. These two methods can be seen as two extremes of a simple approach that assumes the RE distribution must show some kind of statistical dependency with the expression data. The hypergeometric approach is a local criterion, as it considers motif enrichment in a single co-expression cluster, while the mutual information approach is a global one, as it includes contributions from all clusters. Since they make few assumptions, these two methods can be applied to a wide range of expression data, and they are particularly useful when knowledge of the underlying regulatory mechanisms is limited. FIRE and GEMS accurately predicted several of the *P. falciparum *ApiAP2 binding sites (experimentally validated in [17,18]), and FIRE has recently been used to detect RNA recognition elements in *Saccharomyces cerevisiae *[19].

In this work, we show how the hypergeometric distribution (local criterion) and the mutual information (global criterion) can be used without requiring any clustering, using the notion of motif density in the expression space. Rather than considering the number of genes that contain a motif in each co-expression cluster, we estimate motif densities locally around each gene by considering the proportion of neighboring genes (in the expression space) that contain the motif. The remainder of the paper is organized as follows. We first present the continuous versions of the hypergeometric and mutual information criteria, and the RED^2 ^software that implements them. Second, we describe the output of RED^2 ^and explain how the motif density can be used to assess the functionality of a particular motif occurrence. Third, we compare the original and new versions of the two criteria on several expression datasets from yeast, and show that using the new versions usually results in a significant increase in the motif detection sensitivity. In addition, we perform a similar comparison with two well-established methods, FIRE and MatrixREDUCE, which demonstrates the broad utility of our approach. Finally, as a case study, we apply RED^2 ^on two *P. falciparum *datasets and discuss some findings that can be deduced from the results.

## Results and discussion

We are given a set *G *of *n *genes with two kinds of data. First, each gene is associated with a vector that describes its expression level at different time points or in a given set of conditions (that is, its expression profile). Second, each gene is associated with a nucleic acid sequence, which may correspond to its upstream, intronic or downstream region. In addition, we are given a function to compute the distance between the expression profile of two genes - generally, this is the Euclidean or the Pearson correlation distance. As noted in the introduction, the goal is to identify motifs whose presence or absence in the sequences shows statistical dependency with the expression data.

Broadly speaking, a motif *m *is a set of sequences defined over {A, C, G, T}. We say that a sequence *s *contains a motif *m *if at least one substring of *s *belongs to *m*. Two popular ways of representing motifs are the position weight matrices (PWMs) and consensus strings with degenerate symbols (IUPAC system) [5]. Here we use the IUPAC system, representing motifs with special characters encoding alternative nucleic acids; for example, the WAGACA motif corresponds to {AAGACA, TAGACA}. In theory, PWMs are more expressive than IUPAC strings, as they can represent more motifs and associate a score to each possible occurrence. However, PWMs require a score threshold to determine whether a particular sequence is a match or not, which may be hard to set. Moreover, they have been shown to perform similarly to IUPAC strings in a benchmark study [5], and, from a computational perspective, dealing with IUPAC strings is less demanding than PWMs.

### Scoring functions

Here we describe the local and global criteria used by GEMS and FIRE, respectively, and show how they can be used without clustering, by computing motif density locally around each gene in the expression space. Namely, given a gene *g *∈ *G *and a motif *m*, the motif density around *g *is simply the proportion of genes that possess *m *among the neighbors of *g*. This involves defining a neighborhood for each gene in the expression space. We could use the *k*-nearest neighbors of the gene according to the chosen distance function (Euclidean or Pearson), but we will see that using this neighborhood may reduce the sensitivity of the approach because of the hubness phenomenon [20]. Therefore, after presenting the two criteria, we propose an alternative solution to define the neighborhood.

#### Local criterion

The GEMS algorithm expresses the dependency between a motif and the expression data in terms of motif enrichment, assuming that a motif is likely to have a biological function if, in any given co-expression cluster, it is present in more genes than would be expected by chance. Let *c *be a co-expression cluster containing *n_c _*genes and let *n_mc _*denote the number of genes having the motif *m *in *c*. Under the null hypothesis that *m *is distributed independently of the expression data, the probability of observing *X *≥ *n_mc _*positive genes (that is, containing *m*) in *c *is derived from the hypergeometric distribution:

$$P\left(X\geq n_{mc}\right)=\overset{\text{min}\left(n_{c},n_{m}\right)}{\underset{j=n_{mc}}{\sum }}\frac{\left(\begin{array}{c} n_{m}\\ j \end{array}\right)\left(\begin{array}{c} n-n_{m}\\ n_{c}-j \end{array}\right)}{\left(\begin{array}{c} n\\ n_{c} \end{array}\right)},$$

where *n_m _*is the total number of genes with motif *m *in *G*. For a given clustering *X*, the motif enrichment score of *m *is defined in GEMS as:

(1) $$s\left(m,C\right)=\underset{c\in C}{\text{max}}\left(-\text{log}P\left(X\geq n_{mc}\right)\right).$$

This is a local criterion because the score of the motif is given by the single cluster where the enrichment is the most significant.

A straightforward way of modifying this function for non-clustered data is to take each gene *g *∈ *G *in turn, and to consider the number of positive genes (that is, which have the motif) in its neighborhood. Let $n_{mk_{g}}$ denote the number of positive genes among the *k *neighbors of *g*. This leads to:

(2) $$s\left(m,G\right)=\underset{c\in G}{\text{max}}\left(-\text{log}P\left(X\geq n_{mk_{g}}\right)\right).$$

A major difference between Equations (1) and (2) is that the latter considers different sets of overlapping genes, which is biologically more sound than considering a single partition when seeking multiple sets of co-regulated genes. However, an obvious issue with Equation (2) is the multiplication of tests required for each motif. We will see that this is not a problem in practice because this quantity is not utilized as a *P *value but rather as a score function, which is used to compute a false discovery rate.

#### Global criterion

The FIRE algorithm expresses the dependency between a motif *m *and the co-expression clusters in terms of mutual information. Assume that we randomly pick a gene *g *∈ *G *and let *C *be a random variable whose outcome is the cluster membership of *g*. Similarly, let *M *be a random variable whose outcome is 1 if *g *has motif *m*, and 0 otherwise. The mutual information between *M *and *C *is defined in FIRE as:

(3) $$I\left(M;C\right)=\underset{M,C}{\sum }P\left(M,C\right)\text{log}\frac{P\left(M,C\right)}{P\left(M\right)P\left(C\right)},$$

with *P*(*C*) = *n_c_*/*n*. Moreover, *P*(*M*) and *P*(*M*,*C*) are written as:

$$P\left(M\right)=\left\{\begin{array}{c} \frac{n_{m}}{n}\text{, if }M=1\\ 1-\frac{n_{m}}{n}\text{, if }M=0 \end{array}\right)$$

and

$$P\left(M,C\right)=\left\{\begin{array}{c} \frac{n_{m,c}}{n}\text{, if }M=1\\ 1-\frac{n_{m,c}}{n}\text{, if }M=0 \end{array}\right).$$

To get rid of the clustering, let us consider the probability density function over the space of the gene expression profiles. The gene expression profiles are assumed to be sampled from this density. The mutual information between the presence/absence of a motif *m *and a gene expression profile *X *is equal to:

$$\begin{array}{llll} I\left(M;X\right) & =\underset{X}{\int }\underset{M}{\sum }P\left(M,X\right)\text{log}\frac{P\left(M,X\right)}{P\left(M\right)P\left(X\right)}dX\; & & \;\\ & =\underset{X}{\int }P\left(X\right)\left(\underset{M}{\sum }P\left(M|X\right)\text{log}\frac{P\left(M|X\right)}{P\left(M\right)}\right)dX.\; & & \;\\ & \; & \end{array}$$

This expression can be interpreted as the expected value of the function:

$$f\left(X\right)=\underset{M}{\sum }P\left(M|X\right)\text{log}\frac{P\left(M|X\right)}{P\left(M\right)}$$

with respect to the profile density function. Hence, *I*(*M*;*X*) can be estimated from the average:

(4) $$\frac{1}{n}\underset{g\in G}{\sum }\underset{M}{\sum }P\left(M|X_{g}\right)\text{log}\frac{P\left(M|X_{g}\right)}{P\left(M\right)},$$

where *X_g _*is the expression profile of gene *g*. Probability *P*(*M *| *X_g_*) is estimated using the motif density around *g*, that is, the proportion of positive genes among the *k *neighbors of *g*:

$$P\left(M|X_{g}\right)=\left\{\begin{array}{c} \frac{n_{mk_{g}}}{n}\text{, if }M=1\\ 1-\frac{n_{mk_{g}}}{k}\text{, if }M=0 \end{array}\right).$$

#### Neighborhood definition

**Figure 2:**
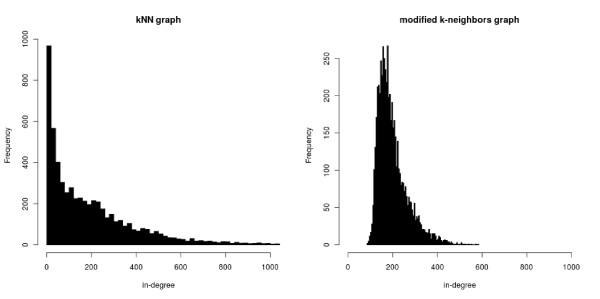
**In-degree distribution of two different neighborhood graphs for the Gasch *et al. S. cerevisiae *gene expression compendium **[21]. (Left) *k *nearest-neighbors graph (*k *= 200). (Right) Our modified *k*-neighbors graph (*k *= 200).

In the above formulae (Equations (2) and (4)), we use the number of genes that contain the motif among the *k *neighbors of each gene. A natural solution to define this neighborhood is to consider the *k*-nearest neighbors (kNN) of the gene. However, a major drawback of this approach stems from the *hubness *phenomenon [20], which occurs in high-dimensional space. This phenomenon can be described as the tendency for a few points (genes) to appear in an excessive number of neighborhoods, while many other genes appear in zero or few neighborhoods. If we encode the kNN relationships into a directed graph by putting an arc from node (gene) *g *to node *g' *if *g' *is among the *k*-nearest neighbors of *g*, we observe that the in-degree distribution of this graph - that is, the number of arcs ending on a node - is strongly skewed to the left. This is exemplified in the left panel of Figure 2, which is obtained with the Gasch *et al. *[21] dataset, a compendium of yeast transcriptomic measurements in a variety of stress conditions (see experiments below). Such a neighborhood biases our overall density estimation, since it gives more weight to the most popular neighbors, while discarding the information provided by the least popular ones. Radovanović *et al. *[20] showed that this phenomenon can affect both supervised (for example, kNN predictor) and unsupervised (for example, k-means) classification algorithms that use neighborhoods. In our case, the effect is particularly important, as it can prevent the discovery of motifs associated with genes that do not belong to the hubs. To circumvent this problem, we have designed a procedure that starts from the kNN graph described above. In this graph, the neighborhood of a gene *g *involves all genes *g' *such that there is an arc *g *→ *g'*. First, the neighborhood relations are symmetrized: if there is an arc *g *→ *g' *and no arc *g' *→ *g*, the latter is added to the graph. In this way, each gene is in the neighborhood of at least *k *other genes. We then use this symmetrized graph to construct a second graph in the following way. For each gene, we select *k *neighbors by randomly sampling without replacement among its neighborhood, using a probability distribution inversely proportional to the number of neighborhoods in which the neighbors appear (see Material and methods for details). Hence, genes that are in many neighborhoods have less chance of appearing in each of these neighborhoods. As we observe in the right panel of Figure 2, this strategy reduces the asymmetry of the in-degree distribution of the neighborhood graph. It would likely be possible to define other procedures to achieve this goal, but, as we will see in the experiments, this one effectively increases the sensitivity of our motif detection approach.

### The RED^2 ^software

The new versions of the scoring functions have been implemented (C++) in software called RED^2 ^(for regulatory element discovery from raw expression data). RED^2 ^takes as input a set of expression profiles and their associated DNA sequences, and outputs a set of IUPAC motifs with various statistics, such as their distribution in the expression space and in the input sequences. To make the analyses easier, the promoter sequences of several model organisms are pre-loaded on the web server. Like many methods for motif discovery, RED^2 ^starts by computing the score of every possible *q*-mer (*q *= 7 by default) and then proceeds with a local optimization of the highest scoring *q*-mers (called the seeds), transforming them into richer and better scoring motifs. When all seeds have been optimized, a filtering step removes the redundancy in the set of inferred motifs. We describe the RED^2 ^implementation and output below.

#### Seed identification step

To assess the significance of a seed score while taking into account multiple testing, we estimate the proportion of false positives (false discovery rate, FDR) for different score thresholds. For this reason, we rank *q*-mers according to their score and compare the resulting list with the expected score distribution under hypothesis *H*_0 _that there is no dependency between *q*-mers and expression profiles. This distribution is estimated by randomly shuffling the mapping between the nucleic acid sequences and the expression profiles, and re-evaluating all *q*-mer scores several times (ten by default). The FDR associated with an observed score *s *is estimated with the formula:

(5) $$\text{FDR}=\frac{\text{Expected number of }q\text{ - mers with a score }\geq s\text{ under }H_{0}}{\text{Number of }q\text{ - mers with a score }\geq s\text{ in original data}}.$$

*q*-mers with a score associated with an FDR below a given threshold (0.001 by default) are then considered for optimization. Note that the above formula may sometimes give FDR > 1. In this case the FDR is assumed to be 1 for the considered score.

#### Optimization step

Each seed is iteratively refined in a greedy way until its score reaches a local maximum. At each iteration, we evaluate the score of all solutions (motifs) obtained by replacing any non-degenerate position of the current solution by any IUPAC symbol generalizing the nucleotide at this position, and the best scoring solution is selected for the next iteration. For example, the motif WAGACA will lead to the set {WWGACA, WHGACA, ..., WARACA, ...}, where W = {A, T}, H = {A, T, C} and R = {A, G}.

**Figure 3:**
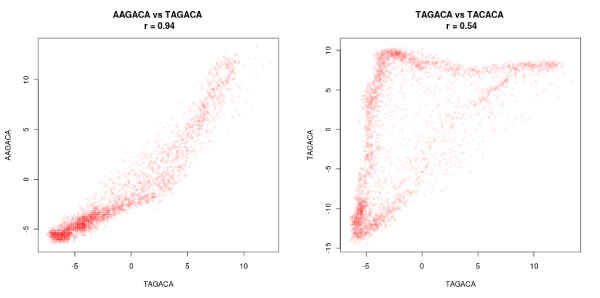
**Comparison between different motifs in *P. falciparum*, using the Pearson correlation between their density profiles **[11]. Each point corresponds to a gene and represents a pair of density estimates (one for each motif). The left figure suggests that the AAGACA and TAGACA sequences belong to the same motif (WAGACA), as their density profiles have a Pearson correlation coefficient of 0.94. In contrast, the TAGACA and TACACA sequences have clearly different density profiles (Pearson coefficient of 0.54), despite the fact that they differ by only one nucleotide, as shown in the right figure. The densities are estimated using 200 neighbors for each gene and are expressed as a z-score (see motif density in Material and methods).

A critical point when generalizing the seeds is to avoid constructing a motif whose sequence description looks similar to that of the seed, but which is present in genes associated with very different expression profiles. To prevent such incoherent motifs, RED^2 ^considers a motif *m *that generalizes a seed *s *as a potential solution only if the genes covered by *m *are close to those covered by *s *in the expression space. For this reason, RED^2 ^uses the density profiles of the motifs, that is, the vector of length *n *that describes the density of the motif around each gene in *G*. Recall that the density of *m *around a gene *g *is the proportion of genes with the motif in the neighborhood of *g*, that is $n_{mk_{g}}/k$. Then, *m *is considered as a potential optimization of *s *only if the Pearson correlation between the density profiles of *m *and *s *is above a fixed threshold α (α = 0.75 by default). This point is illustrated in Figure 3, which compares the density profiles of two pairs of close 6-mers in *P. falciparum *using the Bozdech dataset (see the application to *P. falciparum *below). The 6-mers of the first pair have similar density profiles, suggesting that they are part of the same regulatory motif. In contrast, the 6-mers of the second pair have quite different profiles, suggesting that they are bound by different regulatory proteins.

When this first optimization step has reached a local maximum, RED^2 ^tries to elongate each motif with a similar procedure. In this second step, solutions are obtained by adding any IUPAC symbol to one of the motif extremities. The parameter α is not used here, as this step can only decrease the set of sequences in *m*. The maximum motif length is set at nine by default, but it can be increased up to 15.

#### Filtering step

Several seeds may be associated with the same biological motif, and the optimization step usually leads to a highly redundant set of motifs. To retain the best variant of each motif, RED^2 ^compares them in two ways. First, it computes the overlap between each pair of motifs. The overlap is defined as the maximum number of positions that can be aligned without internal gaps or mismatches (two IUPAC symbols match if their intersection is non-empty). For example, the overlap between WAGACA and TATAGA is four, corresponding to the alignment:

$$\begin{array}{cccccccc} - & - & \text{W} & \text{A} & \text{G} & \text{A} & \text{C} & \text{A}\\ \text{T} & \text{A} & \text{T} & \text{A} & \text{G} & \text{A} & - & - \end{array}$$

By default, RED^2 ^considers that two motifs may be redundant if their overlap is at least four. However, as discussed above, similar motifs (at the sequence level) may be associated with different locations in the expression space, and hence correspond to different transcription factors. Therefore, to be considered redundant, the Pearson correlation between their density profiles must also be above a fixed threshold γ (γ = 0.75 by default).

The filtering step proceeds as follows. All optimized seeds are sorted according to their score and the best scoring one is put in the (empty) set of filtered motifs. Then the remaining ones are considered sequentially and added to the set of filtered motifs if they are not redundant with respect to that set.

#### RED^2 ^output and functionality of motif occurrences

**Figure 4:**
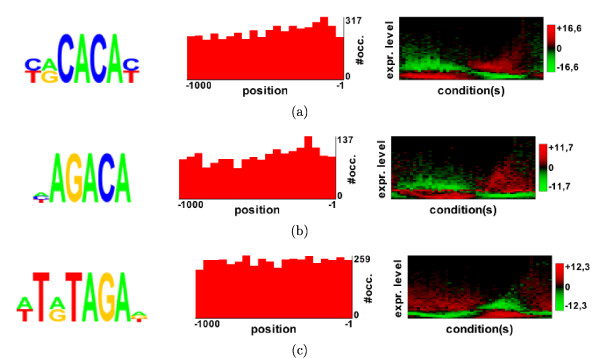
**The three highest scoring motifs found in the Bozdech analysis **[11]. (Left) Motif logos. (Middle) Distance distributions according to the start codon. (Right) Profile heatmaps. The x-axis of this map represents the different conditions in the expression dataset, while the y-axis represents the expression level. The y-axis is divided into 25 bins, and the colors of the map indicate the motif density (expressed as a z-score) in each of these bins (red: high density; green: low density).

For each motif, RED^2 ^returns the logo of the motif together with various statistics and graphics (see Figure 4). Firstly, a histogram describes the distribution of motif occurrence positions relative to the 5' or 3' sequence extremities (see middle of Figure 4). Secondly, a heatmap (right of Figure 4) shows the distribution of the motif in the expression space. The x-axis of this map represents the different conditions in the expression dataset, while the y-axis represents the expression level. The y-axis is divided into 25 bins, and the colors of the map indicate the motif density in each of these bins, expressed as a z-score (red for high and green for low; see motif density in Material and methods). For example, in Figure 4, which shows three motifs identified in *P. falciparum *from the Bozdech dataset (see the application to *P. falciparum *below), we can see that the first motif (Figure 4a) is overrepresented in genes that have low expression levels in the first third of the conditions (that is, in the first 16 h of the erythrocytic cycle), high expression levels in the following hours, and again low levels in the last few hours. In contrast, the third motif (Figure 4(c)) is overrepresented in genes that have quite opposite profiles.

**Figure 5:**
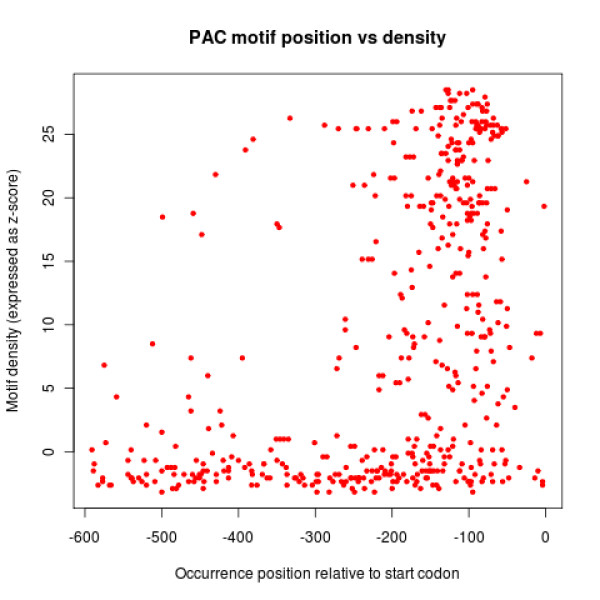
**Relationship between motif density and functionality**. Each point corresponds to a PAC/TOD6 motif occurrence (HGMGATGAG) in a promoter region of a *S. cerevisiae *gene. The x-axis represents the distance in bp of an occurrence from the start codon. The y-axis represents the motif density around the gene where the occurrence is found. Density is estimated by considering the number of genes containing the PAC motif in the neighborhood of each gene (Gasch *et al. *dataset) [11], and is expressed as a z-score (see motif density in Material and methods).

For each motif, RED^2 ^uses a sign test to assess a potential strand bias (that is, if the motif occurs preferentially on the forward or reverse strand), and provides a gene ontology (GO) enrichment analysis of the genes possessing the motif. Moreover, it outputs the position and context (10 bp on each side) of all occurrences, and the list of genes that possess the motif, sorted by motif density (z-score). This sorted list is of high practical interest, because the motif density around a gene where a particular occurrence is found can be a good indicator of that occurrence's functionality. The rationale is that a motif occurrence is more likely to be functional in a gene that is in a region of the expression space where many genes also possess the motif. This is illustrated in Figure 5 with the PAC/TOD6 motif, whose functionality is highly constrained by its position in the *S. cerevisiae *promoter sequences [22]. In this figure, the position of all PAC motif occurrences are plotted against their local density with respect to the Gasch *et al. *dataset (see the experiments below). We can see a strong positional bias for occurrences in high motif density regions, while occurrences in low density regions are more uniformly distributed. Similar results are obtained with the SFP1 and RPN4 motifs.

This discriminative feature can be used to refine the selection of genes that are likely regulated by a given motif. To account for the prior probability of each motif, and to better distinguish between high and low density values, RED^2 ^actually expresses the densities as a z-score, which is the difference between the fraction of genes that possess the motifs and the expected value under the null hypothesis, in terms of the standard deviation (see Formula 6 in Material and methods). Thus, positive z-scores indicate genes in regions of the expression space where the proportion of genes that have the motif is higher than expected, while negative z-scores indicate lower than expected proportions.

### Assessment for yeast

We assessed our approach on yeast, which is likely the best known eukaryote for transcriptional regulation. We used 24 expression datasets: 22 datasets from the Gasch *et al. *[21] compendium, the complete compendium itself and the Spellman *et al. *[23] dataset (see Material and methods). The 600 bp upstream of each gene was used in these experiments.

#### Hubness phenomenon

**Figure 6:**
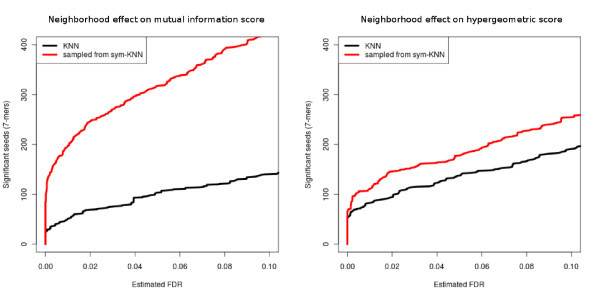
**Effect of the neighborhood definition on the scoring function applied to the Gasch *et al. *dataset **[21]. The y-axis is the number of significant seeds (7-mers) detected at different FDR thresholds (x-axis). (Left) Mutual information scoring function. (Right) Hypergeometric scoring function.

First, we assessed the effect of the hubness phenomenon on the new version of the scoring functions. We mentioned previously that the *k*-nearest neighborhood is subject to this phenomenon, and we proposed an alternative neighborhood based on the symmetric *k*-nearest neighbors. To measure how this affects the performance of our approach, we compared the number of 7-mers detected at different FDR thresholds using both types of neighborhood. Figure 6 shows the results obtained with a neighborhood of size 200 on the Gasch *et al. *dataset [21] (complete compendium). As we can see in this figure, the hubness phenomenon can have a huge effect, especially for the mutual information scoring function, and a proper definition of the gene neighborhood is crucial to achieving good results.

#### Seed identification

We compared the original and continuous versions of the scoring functions on the 24 yeast expression datasets. The clustering used for the original scoring functions was obtained with the k-means algorithm of the R project [24]. For each dataset, we tried several cluster numbers: 5, 10, 20, 40 and 80. For each number, the k-means algorithm was run 100 times and the best clustering (according to the k-means criterion) was retained for the experiments below. For the continuous version of the scoring functions (Equations (2) and (4)), we always used a neighborhood of size 200. Both the clustering and the neighborhood definition were performed using the Euclidean distance between the expression profiles.

**Figure 7:**
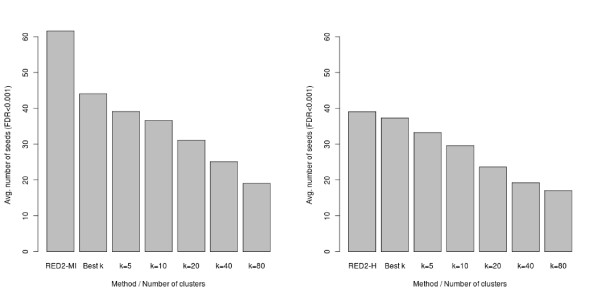
**Average number of seeds detected by the continuous and discrete versions of each scoring function for the 24 yeast datasets (FDR < 0.001)**. (Left) Mutual information. (Right) Hypergeometric. For each panel, the leftmost column shows the results obtained by the continuous version implemented in RED^2^. The five rightmost columns show the results obtained by the discrete version for different numbers (*k*) of clusters. The 'best *k*' column shows the average results when the clustering that yields the highest number of detected seeds is selected *a posteriori *for each dataset.

To focus on the scoring functions and limit the impact of the optimization parameters, we first compared the number of seeds (7-mers) detected at a given FDR threshold by each scoring function. Figure 7 shows the results achieved for different number of clusters (*k*), using an FDR cutoff of 0.001. For comparison, we also include the number of seeds obtained by selecting *a posteriori *the number of clusters that yields the highest number of significant seeds for each dataset (the best *k *column). Note that this selection procedure (hereafter denoted as the best *k *procedure) gives a clear advantage to the original functions, since we did not allow such *a posteriori *selection of the parameters for our new scoring functions. Despite that, we observe that the new functions detect more seeds (at the same FDR) than the original ones, especially for the mutual information criterion. This shows the higher sensitivity of our approach. We also observe that the number of seeds identified by the original scoring functions strongly depends on the number of clusters *k*, with fewer significant seeds for the highest *k *values.

#### Quality of predicted motifs

We considered the motifs we obtained from the seeds after applying the optimization and filtering procedure described above. To evaluate the quality of these motifs, we systematically compared them to known motifs from three curated databases: JASPAR (177 motifs) [25], the collection of Gordân *et al. *(189 motifs) [26] and ScerTF (196 motifs) [27]. To determine if a predicted motif matches a known motif, we used an approach similar to that of the Tomtom algorithm [28]. Briefly, given two aligned motifs, a similarity score is computed by summing (over each aligned position) the Pearson correlation coefficient between the weights of the corresponding columns in the PWMs of the corresponding motifs. For our predicted motifs, 'A' is converted to [1,0,0,0], 'W' to [0.5,0.5,0,0], etc. Using a shuffling procedure that exchanges the PWM columns of the curated database, we then estimate the *P *value of obtaining an alignment of equal or higher score by chance. The best match of a predicted motif is the motif from the database that gives the highest scoring alignment, and hence the lowest *P *value.

To allow a fair comparison between different motif discovery methods, we cannot strictly consider the number of predicted motifs that have a match under a given *P *value, since a method which simply enumerates all the motifs would then achieve the best possible results. Hence, for each method, we took the number of predicted motifs into account and determined how many matches are significant at a global FDR threshold of 15%, using the procedure of Benjamini and Hochberg [29]. This way, each prediction has an underlying cost, and the exhaustive enumeration method or a random predictor would get no significant match.

Another important issue that must be considered is the redundancy among the predictions, since a method which predicts (correctly) multiple variants of the same motif would get an unfair advantage over the other ones. Hence, when the best matches of two or more motifs correspond to the same motif in the database, only one match is considered (that is, the other motif is considered as having no match).

**Figure 8:**
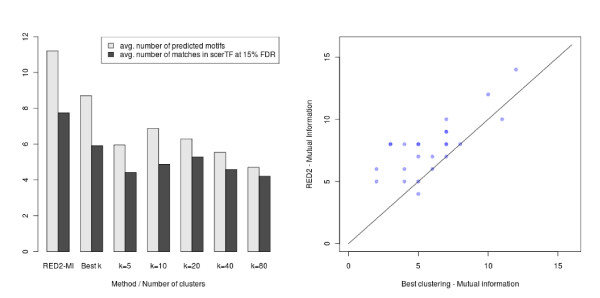
**Average number of motifs and matches at 15% FDR recovered by the continuous and discrete mutual-information scoring functions**. The same optimization and filtering procedure was applied on both versions (seed FDR < 0.001, α = 0.75, γ = 0.75). (Left) Average results for the 24 yeast datasets. The leftmost column shows the results obtained by the continuous version of the scoring function implemented in RED^2^. The '*k *= *i*' columns show the results obtained by the discrete version and *i *clusters. The 'best *k*' column shows the results obtained when the number of clusters that yields the highest number of motifs is selected *a posteriori *for each dataset. (Right) Number of predicted motifs that match a known motif in the ScerTF database for the 24 yeast datasets (FDR < 15%). Each point corresponds to one of the 24 datasets. The y-axis corresponds to the number achieved by RED^2 ^and the x-axis to the number achieved by the discrete version and the best *k *procedure. Superimposed points are indicated by shading. RED^2 ^found more motifs than the best clustering for 18 datasets and fewer for two datasets, which gives a sign test *P *value of 0.0004.

**Figure 9:**
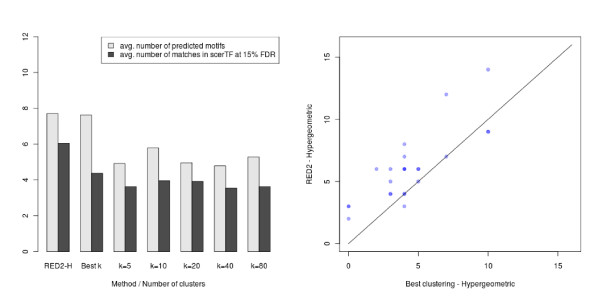
**Average number of motifs and matches at 15% FDR recovered by the continuous and discrete hypergeometric-distribution scoring functions**. The same optimization and filtering procedure was applied on both versions (seed FDR < 0.001, α = 0.75, γ = 0.75). (Left) Average results for the 24 yeast datasets. The leftmost column shows the results obtained by the continuous version of the scoring function implemented in RED^2^. The '*k *= *i*' columns show the results obtained by the discrete version and *i *clusters. The 'best *k*' column shows the results obtained when the number of clusters that yields the highest number of motifs is selected *a posteriori *for each dataset. (Right) Comparison of the number of predicted motifs that match a known motif in the ScerTF database for the 24 yeast datasets at 15% FDR. The y-axis corresponds to the number achieved by RED^2 ^and the x-axis to the number achieved by the discrete version and the best *k*. Superimposed points are indicated by shading. RED^2 ^found more motifs than the best clustering for 17 datasets and fewer for three datasets, which gives a sign test *P *value of 0.003.

In Figure 8 and Figure 9, we present the results obtained with ScerTF, which is the most recent and most complete database of *S. cerevisiae *motifs to date. Results obtained with the two other databases support the same conclusions and are available in Additional file 1. The left panel of these figures presents the average results for the 24 yeast datasets. Each column has two bars: the average number of predicted motifs and the average number of matches at 15% FDR. As we did for the seeds, we also included the results obtained by selecting *a posteriori *the number of clusters *k *that gives the highest number of motifs for each dataset (the best *k *procedure). On average, the continuous versions of the scoring functions substantially predict more motifs than the discrete versions for any number *k *of clusters. More importantly, the continuous versions also have more matches in ScerTF than the discrete versions, at 15% FDR. To determine the significance of the observed gain, we performed a sign test between the number of matches obtained by RED^2 ^and the best *k *procedure for the 24 datasets (see the right panel of Figure 8 and Figure 9). The *P *values of these tests are 0.0004 for the mutual information and 0.003 for the hypergeometric distribution, in favor of RED^2^.

When comparing Figure 7 with Figure 8 and Figure 9, we observe that detecting more seeds does not necessarily lead to more motifs and more significant matches. For example, the highest average number of seeds were obtained with the *k *= 5 clusters for both scoring functions, while the highest average number of predicted motifs and matches at 15% FDR were obtained with *k *= 10 or *k *= 20. This indicates that the number of detected seeds alone cannot be used as a reliable measure of performance for comparing different motif discovery approaches.

#### Comparison with FIRE and MatrixREDUCE

**Figure 10:**
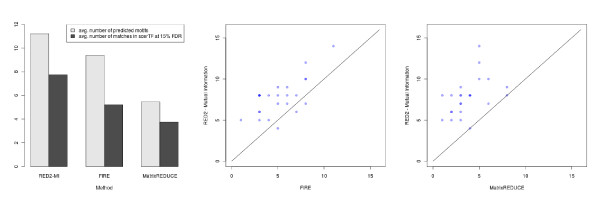
**Number of predicted motifs and number of matches in ScerTF at 15% FDR, for RED^2 ^(mutual-information scoring function; same as Figure 8), FIRE and MatrixREDUCE**. FIRE was run with default parameters (optimized for yeast), with *k *= 5, 10, 20, 40 and 80 clusters, and the number of clusters that yields the highest number of motifs was selected *a posteriori *for each dataset. MatrixREDUCE was run with default parameters, with seeds of length 7, 8 and 9. (Left) Average results of the three methods on the 24 yeast datasets. (Center) RED^2 ^and FIRE. Number of predicted motifs that match a known motif at 15% FDR in the ScerTF database for the 24 yeast datasets. The y-axis corresponds to the number achieved by RED^2 ^and the x-axis to the number achieved by FIRE with the best clustering procedure. Superimposed points are indicated by shading. RED^2 ^has more matches than FIRE in 21 datasets and fewer in three datasets, which gives a sign test *P *value of 0.0003. (Right) RED^2 ^and MatrixREDUCE (same explanations as for the center panel). RED^2 ^has more matches than MatrixREDUCE for 22 datasets and is on par for the remaining two, which gives a sign test *P *value of 4.77 × 10^-7^.

We performed a similar analysis to compare RED^2 ^(with the mutual information scoring function) to the well-established FIRE and MatrixREDUCE software on the same 24 datasets. Both methods were run with default parameters. For FIRE, this corresponds to a seed length of 7 bp and a maximal motif length of 9 bp, as for RED^2^, and we used the best *k *procedure described above. MatrixREDUCE does not allow seed elongation as in RED^2 ^or FIRE, so we performed an optimization of all seeds of length 7, 8 and 9 bp. In both cases, we performed a double strand analysis (that is, without strand distinction). Results are presented in Figure 10. First, we observe (left panel) that RED^2 ^predicts substantially more motifs, on average, than the two other methods. Second, we observe that the number of predictions that match a known motif in ScerTF at 15% FDR is significantly higher for RED^2 ^(7.75) than for FIRE (5.21) and MatrixREDUCE (3.75). This represents a 49% increase over FIRE, and a 206% increase over MatrixREDUCE. A sign test performed on the number of matches obtained for each of the 24 datasets (right panels) gives *P *values of 0.0003 and 4.77 × 10^-7^, for RED^2 ^against FIRE and MatrixREDUCE, respectively.

Note that the results of the above comparison should be interpreted with caution, since the three methods use different procedures to optimize the motifs, filter the output and even control the number of seeds that are optimized. For example, FIRE uses conditional mutual information to optimize each motif with regard to the already optimized motifs, while RED^2 ^independently optimizes each motif. For MatrixREDUCE, the differences are even greater since it is based on a linear regression model that takes the number of motif occurrences in each sequence into account, while RED^2 ^and FIRE consider only the absence or presence of a motif in each sequence. Thus, it is not possible to state objectively the proportions of the observed differences between the output of these programs, which are due to the scoring functions, the clustering step (for FIRE) or the optimization procedures and parameters. Nevertheless, this comparison may be of interest to users who are primary concerned with the number and quality of the predicted motifs.

The motifs predicted by RED^2 ^on the Gasch *et al. *(complete compendium) and the Spellman *et al. *datasets with both scoring functions are provided in Additional files 2 to 5. For comparison, we also provide the list of motifs predicted by FIRE and MatrixREDUCE on the Spellman *et al. *dataset in Additional files 6 and 7. For each motif, these files give the most significant match in the ScerTF database (if any at 15% FDR) and the most overrepresented GO term for the genes that possess the motif.

#### Orphan motifs

All considered methods predicted some motifs that do not match any known motif according to our statistical criteria. These absences should be interpreted with caution, because determining whether a particular motif matches another motif is prone to error [28], and often relies on arbitrary thresholds or criteria. Moreover, a high similarity between two motifs does not always translate into a significant match (and vice versa), especially when the motifs are short and the query database contains many of them. In many cases, careful inspection suggests that these orphan motifs are sub-optimal variants of known motifs.

For example, this is clearly the case with motif HVGGDGGCR, obtained with the mutual information scoring function on the Spellman *et al. *dataset (motif #7 in Additional file 4). This motif roughly corresponds to a 3 bp shift of the motif GKGGCRAMW (motif #8), which significantly matches the RPN4 motif. In other cases, additional experiments are needed to determine if they are new biological motifs or simply false positives. For example, motif CCCTTTWHH (motif #6) does not correspond to any motif from the three considered databases. However, other information suggests that this motif may not be a false positive. First, it is strongly conserved in *S. bayanus *orthologous genes [30]. Second, the set of genes containing this motif is enriched with the lipid modification annotation in the gene ontology (*P *< 1.49 × 10^-2^, Bonferonni corrected).

#### Complementarity of the methods

It is worth noting that the different methods usually predicted complementary sets of motifs. For example, on the Spellman *et al. *dataset, the mutual-information and the hypergeometric scoring functions each recovered nine motifs from the ScerTF database at 15% FDR (Additional files 4 and 5). However, only four motifs are recovered by both methods (MBP1, TOD6, SFP1 and RPN4). The hypergeometric scoring functions uniquely finds GIS1, GCN4, PHO4, REI1 and STE12, while the mutual information uniquely finds YDR026C, RAP1, MSN2, SPT15 and NDT80 (note however that the GIS1 and MSN2 motifs are very similar). On the same dataset, FIRE and MatrixREDUCE find five and eight motifs at 15% FDR, respectively (Additional files 6 and 7). Among them, one (GIS1) and four (STB1, FKH1, RTG3 and STE12) are not recovered by RED^2 ^(mutual information), respectively. Importantly, the complementarity of the methods is not restricted to individual datasets.

Table 1 shows, for each pair of methods (*m*_1_, *m*_2_), the number of ScerTF motifs that are recovered in at least one of the 24 datasets by *m*_1_, but never by *m*_2_. For example, among the 37 motifs recovered by RED^2 ^and the hypergeometric scoring function, 13 are not recovered by FIRE.

**Table 1:** Number of motifs recovered and not recovered by the different methods at 15% FDR

	Total number of motifs recovered	RED2-MI	RED2-H	FIRE	MatrixREDUCE
RED2-MI	45	-	21	21	35
RED2-H	37	13	-	13	25
FIRE	39	15	15	-	28
MatrixREDUCE	20	10	8	9	-

We observe, for every pair of methods, that the number of uniquely identified motifs is an important fraction of the total number of recovered motifs. As expected, RED^2 ^with the mutual-information criterion uniquely identifies the highest number of motifs. However, FIRE also identifies several motifs that are not recovered by the other approaches (among 39 recovered motifs, 15 are not recovered by RED^2 ^with mutual information, and 28 are not recovered by MatrixREDUCE). Moreover, although MatrixREDUCE recovers substantially less motifs (20) than the other approaches, the recovered motifs are often unique to this approach. A careful inspection suggests that these motifs often belong to a small set of genes that do not have correlated expression profiles but are over- or under-expressed in one or a few particular experiments. We provide in Additional file 8 the number of datasets in which each ScerTF motif was recovered at 15% FDR (only the motifs recovered by at least one method are shown).

In the experiments above, we considered the number of predictions that match a known motif for a given global FDR. Since this approach takes the number of predictions into account, it is essential to have a nearly complete collection of known motifs. Otherwise, the FDR would be overestimated and many good predictions would have no significant match. In consequence, we restricted our benchmark analysis to yeast, which is the only eukaryote for which such collections exist. Nevertheless, we also applied RED^2 ^to several apicomplexan parasites (see the application to *P. falciparum *below), as well as to human and *Arabidopsis thaliana*. For this latter, RED^2 ^has been successfully used to detect DNA motifs involved in transcriptional regulation following phosphate starvation and specific drug treatment [31].

#### Application to *P. falciparum*

*P. falciparum*, the main causal agent of human malaria, kills nearly 800,000 people yearly in the 106 malaria-endemic countries [32]. Its genome is very atypical: around 60% of the approximately 5,500 predicted genes have insufficient similarity to characterized genes in other species to justify functional assignment [33]. The way the parasite controls the expression of its genes is also poorly understood. While several microarray studies have revealed intricate and tight gene expression regulation, we are unaware of the mechanisms underlying this control, and, more specifically, of the relative role of transcriptional and post-transcriptional regulation in the parasite [34]. Moreover, with the notable exception of the recently discovered ApiAP2 domain [35], most attempts to identify transcriptional factors in *P. falciparum *have failed.

As a case study, we ran RED^2 ^on the datasets of Bozdech *et al. *[11] and Shock *et al. *[36]. The former measured mRNA levels of *P. falciparum *genes once per hour during one complete 48 h intraerythrocytic cycle, while the latter measured mRNA-decay profiles during this same cycle. For each dataset, we performed two separate analyses. In the first, we searched for putative motifs involved in mRNA synthesis regulation, that is, located up to one kilobase upstream of the start codon, either on the forward or the reverse strand. In the second, we searched for motifs that are likely involved in mRNA degradation or sequestration, that is located up to 500 bp downstream of the stop codon, on the forward strand only, which approximately corresponds to the 3'UTR. RED^2 ^was run with the mutual information scoring function, and the following parameters: α = 0.9, γ = 0.75, FDR threshold = 0.001. For the downstream analysis, we also increased the maximum motif length to 12. Complete results for these analyses are provided in Additional files 9 and 10.

The upstream analysis of the Bozdech gene expression dataset yields a total of 21 motifs. Eight of these motifs match (at 15% FDR) one of the 23 ApiAP2 motifs previously identified in a protein binding microarray experiment [18]. Among the motifs that do not match any ApiAP2 motifs, the HAGACA motif (see Figure 4b) achieves a very high score (it is the second best scoring motif of the analysis) and may be the binding site of a yet uncharacterized transcription factor. Downstream analysis on the same dataset identified no motifs. FIRE and MatrixREDUCE (with default parameters) get six and three matches (at 15% FDR) on this dataset, respectively (see Additional files 11 and 12).

**Figure 11:**
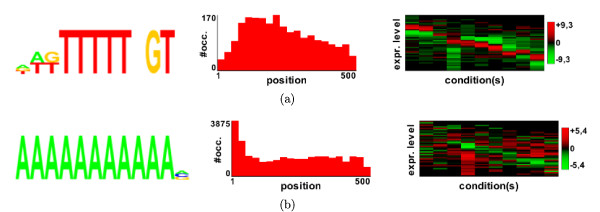
**The two highest scoring motifs found in the Shock analysis, using the mutual information scoring function**. (Left) Motif logos. (Middle) Distance distributions according to the stop codon. (Right) Profile heatmaps.

In contrast, downstream analysis of the Shock mRNA decay dataset returns six motifs. The best scoring one (HWKTTTTTNGT, see Figure 11a) is partially included in a 47 bp motif previously identified in the 3'UTR of nine translationally repressed transcripts [37], and is very similar to a motif (TYTTTTNGT) identified in the downstream region of some *P. falciparum *genes by comparative genomics with *P. yoelii *and *P. knowlesi *[30]. To the best of our knowledge, the second best scoring motif (AAAAAAAAAAAV, Figure 11b) does not resemble any other motif already described in the literature. When looking at the occurrence position distributions, we see that both motifs have a high, although very different, positional bias. We can also see that the genes where these motifs are present have quite opposite mRNA decay profiles, and that these two motifs seem to define two different gene classes. The three remaining motifs achieve lower scores, and look similar to the best two motifs, being either T- or A-rich. Moreover, a visual inspection of their position distribution in the sequence and in the expression space shows that they are also similarly distributed. Hence, it is tempting to speculate that these motifs are variants of the best two motifs, or are complementary motifs working together with the latter.

Interestingly, a downstream analysis with the Bozdech dataset did not return any motifs. Hence, the fact that these motifs were identified only in the downstream analysis of the Shock dataset supports the hypothesis that they are involved in mRNA degradation or sequestration. Moreover, it is worth noting that the downstream analysis discovered few motifs (five) compared with the equivalent upstream analysis (> 20). Together, these results seem to indicate that either transcript regulation in *P. falciparum *mainly occurs at the mRNA synthesis level, or that important downstream motifs cannot be represented as consensus sequences (for example, structural motifs).

The RED^2 ^analysis results for yeast and *P. falciparum *have several differences. In *S. cerevisiae*, many motifs show a strong positional bias towards a small region located approximately 150 bp upstream of the genes (for example, SFP1, TOD6, MBP1, YDR026C and STB1, see Additional files 2 to 5). Interestingly, this kind of positional bias is not observed in the upstream analysis of *P. falciparum*, where motif occurrences are more uniformly distributed (see for example Additional file 9). Moreover, there is a strong strand bias for many motifs found in *P. falciparum - *the *P *value of the sign test is below 10^−3 ^for six motifs - but very few in *S. cerevisiae*. Finally, we note that the average number of motif occurrences per gene is significantly higher in *P. falciparum *than in *S. cerevisiae*. Overall, these results may reflect marked differences between the gene regulation mechanisms in the two species.

## Conclusions

Most approaches for *in silico *discovery of regulatory elements require clustering of the expression data. This implicitly assumes that genes belong to disjoint classes, an assumption that rarely holds in the regulation context. In this paper, we have presented a new approach for discovering regulatory elements that does not require clustering and allows overlaps between different gene sets. This is done by estimating motif densities locally around each gene using a fixed-size neighborhood. We have adapted the hypergeometric and mutual-information criteria to this approach, and have shown for yeast that the new versions outperform the original methods, both in terms of statistical sensitivity and the number of experimentally validated motifs they retrieve. A comparison with the FIRE and MatrixREDUCE algorithms also demonstrated the complementarity of the different approaches.

Furthermore, we have shown that motif densities in the gene-expression space are useful for comparing and discriminating between different motifs. This discriminative feature is extensively used in our optimization procedure to avoid merging motifs whose descriptions look similar at the sequence level, but which are associated with different locations in the expression space. Similarly, the density of a motif around a particular gene can help to predict if an occurrence of this motif is biologically active or not.

The scoring functions and methods presented in this paper have been implemented in the RED^2 ^software [38]. RED^2 ^returns the identified motifs along with other information and has a user-friendly interface. A RED^2 ^analysis usually takes between 5 and 20 min, depending on the number of screened genes and the size of the analyzed sequences.

There are several enhancements for this work. First, we intend to enrich the RED^2 ^server with additional pre-loaded species and tools. For example, we plan to add a motif comparison tool that would make use of both DNA sequence and gene expression to compute a similarity score between two motifs, in a way similar to that of our filtering procedure. Finally, another interesting enhancement would be to supplement RED^2 ^with information on close species in order to improve both its sensitivity and accuracy.

## Material and methods

### Neighborhood definition

Let *Γ *be the *k*-nearest neighbor graph obtained by putting an arc from the node (gene) *g *to the node *g' *if *g' *is among the *k*-nearest neighbors of *g*. In this graph, the neighborhood of a gene *g *involves all genes *g' *such that there is an arc *g *→ *g'*. Firstly, the neighborhood relations are symmetrized: if there is an arc *g *→ *g' *and no arc *g' *→ *g*, the latter is added to the graph. This gives us a new graph *Γ*∗. In this graph, each gene is in the neighborhood of at least *k *other genes. We use *Γ*∗ to progressively construct *Γ_k_*, a directed graph where each gene has exactly *k *neighbors, in a way that reduces the skewness of the in-degree distribution. More precisely, the *k *neighbors of each gene *g *are successively sampled from its neighbors in *Γ*∗, according to the following distribution:

$$P\left(g^{\prime }\right)={\left[\underset{g^{\prime \prime }\in N\left(g\right)}{\sum }\frac{d\left(g^{\prime }\right)}{d\left(g^{\prime \prime }\right)}\right]}^{-1},$$

where *d*(*g'*) is the in-degree of gene *g' *in *Γ*∗, and *N*(g) the set of neighbors of *g *in *Γ*∗ that have not yet been included in *Γ_k_*. In other words, the probability for a gene to be chosen as a neighbor is inversely proportional to the number of times it can be chosen.

### Motif density

The density of a motif in a subset of genes is simply the proportion of genes that contain the motif in that subset. To better distinguish between high and low density values, it is sometimes convenient to express the density as a z-score. Let *n_m _*be the number of genes that contain a given motif *m *among a total of *n *genes. Under *H*_0 _(random motif distribution), the number of genes that contain the motif among a sample of *k *genes (*n_mk_*) follows a hypergeometric distribution [39] of parameters *n*, *n_m _*and *k*. Thus, the z-score of an observed density is given by:

(6) $$\frac{kn_{m}/n}{\sqrt{\frac{kn_{m}\left(n-k\right)\left(n-n_{m}\right)}{n^{2}\left(n-1\right)}}}.$$

This quantity is positive (respectively negative) if the number of genes that possess the motif in the sample is higher (respectively lower) than expected.

### Yeast and *P. falciparum *data

The 1 kb and 600 bp upstream sequences of *P. falciparum *and *S. cerevisiae *(respectively) were downloaded from the FIRE website [6]. The 500 bp downstream sequences of *P. falciparum *were downloaded from the PlasmoDB database [40] (version 6.3). To prevent any bias that could result from multi-gene families [6], paralogs were identified using OrthoMCL [41] and discarded.

The Gasch *et al. *[21] compendium was divided into 22 subsets according to the descriptions available in the file. The resulting partition is available in Additional file 13. Each dataset then has expression profiles of between 3 to 15 time points/experiment, with an average number of 7.5 time points/experiment per dataset.

## Abbreviations

FDR: false discovery rate; GO: gene ontology; kNN: *k*-nearest neighbors; PWM: position weight matrix; RE: regulatory element; RED^2^: regulatory element discovery from raw expression data.

## Competing interests

The authors declare that they have no competing interests.

## Authors' contributions

ML and LB designed the method and experiments, and drafted the manuscript. ML conceived and implemented the program and conducted the experiments. OG suggested method improvements and revised the manuscript. VL integrated the program into the web server and made technical improvements. LB coordinated the study. All authors read and approved the final manuscript.

## Supplementary Material

Additional file 1 **Assessment for *S. cerevisiae *using the JASPAR and the Gordân *et al. *databases**. Results obtained when using the JASPAR and the Gordân *et al. *databases (instead of ScerTF). This corresponds to the experiments shown in Figures 8 to 10 in the main manuscript. Click here for file

Additional file 2 **Results of RED^2 ^with mutual information criterion on the *S. cerevisiae *upstream regions with the Gasch *et al. *stress compendium**. The set of motifs inferred by RED^2 ^on the Gasch *et al. *complete compendium. Provided for each motif are the logo of the motif, the number of genes that have the motif in their promoter region, the expression profiles (heatmap) associated with the motif, a histogram of the motif occurrence positions, the existence/absence of any strand bias, and the name of the ScerTF motif that best matches the inferred motif (if any at 15% FDR). Click here for file

Additional file 3 **Results of RED^2 ^(hypergeometric) on *S. cerevisiae *upstream regions with the Gasch *et al. *stress compendium**. The set of motifs inferred by RED^2 ^on the Gasch *et al. *complete compendium. See the description of Additional file 2 for table column definitions. Click here for file

Additional file 4 **Results of RED^2 ^(mutual information) on *S. cerevisiae *upstream regions with the Spellman *et al. *cell-cycle dataset**. The set of motifs inferred by RED^2 ^on the Spellman *et al. *dataset. See the description of Additional file 2 for table column definitions. Click here for file

Additional file 5 **Results of RED^2 ^(hypergeometric) on *S. cerevisiae *upstream regions with the Spellman *et al. *cell-cycle dataset**. The set of motifs inferred by RED^2 ^on the Spellman *et al. *dataset. See the description of Additional file 2 for table column definitions. Click here for file

Additional file 6 **Results of FIRE on *S. cerevisiae *upstream regions with the Spellman *et al. *cell-cycle dataset**. The set of motifs inferred by FIRE on the Spellman *et al. *dataset. See the description of Additional file 2 for table column definitions. Click here for file

Additional file 7 **Results of MatrixREDUCE on *S. cerevisiae *upstream regions with the Spellman *et al. *cell-cycle dataset**. The set of motifs inferred by MatrixREDUCE on the Spellman *et al. *dataset. See the description of Additional file 2 for table column definitions. Click here for file

Additional file 8 **ScerTF motifs recovered by RED^2^, FIRE and MatrixREDUCE on the 24 *S. cerevisiae *datasets**. For each method, this table provides the number of datasets in which each motif was recovered (at 15% FDR). For example, REDUCE correctly predicts AFT1 (first line) in one dataset. The last two lines give the total number of correct predictions (Total) and the number of distinct motifs recovered (Coverage). For example, for the 24 datasets, RED^2 ^makes 177 correct predictions, which cover 43 distinct motifs in ScerTF. Click here for file

Additional file 9 **Results of RED^2 ^(mutual information) on *P. falciparum *upstream regions with the Bozdech *et al. *dataset (erythrocytic cycle)**. The set of motifs inferred by RED^2 ^on the upstream regions of *P. falciparum *genes using the Bozdech *et al. *dataset [11]. See the description of Additional file 2 for a description of the different columns. Click here for file

Additional file 10 **Results of RED^2 ^(mutual information) on *P. falciparum *downstream regions with the Shock *et al. *dataset (mRNA decay)**. The set of motifs inferred by RED^2 ^on the downstream regions of *P. falciparum *genes using the Shock *et al. *dataset [36]. See the description of Additional file 2 for table column definitions. Click here for file

Additional file 11 **Results of FIRE on *P. falciparum *upstream regions with the Bozdech *et al. *dataset (erythrocytic cycle)**. The set of motifs inferred by FIRE on the upstream regions of *P. falciparum *genes using the Bozdech *et al. *dataset [11]. See the description of Additional file 2 for table column definitions. Click here for file

Additional file 12 **Results of MatrixREDUCE on *P. falciparum *upstream regions with the Bozdech *et al. *dataset (erythrocytic cycle)**. The set of motifs inferred by MatrixREDUCE on the upstream regions of *P. falciparum *genes using the Bozdech *et al. *dataset [11]. See the description of Additional file 2 for table column definitions. Click here for file

Additional file 13 **The 22 datasets from the Gasch *et al. *compendium**. This text file describes the partition of the Gasch *et al. *[21] stress compendium into 22 test datasets. Click here for file
