# Supplementary material for: Computational discovery of regulatory elements in a continuous expression space
Source: Genome Biol. 2012 Nov 27;13(11):R109. doi: 10.1186/gb-2012-13-11-r109 (PMC4053739; doi:10.1186/gb-2012-13-11-r109)
Supplement: Additional file 1 — Assessment for S. cerevisiae using the JASPAR and the Gordân et al. databases. Results obtained when using the JASPAR and the Gordân et al. databases (instead of ScerTF). This corresponds to the experiments shown in Figures 8 to 10 in the main manuscript. [file gb-2012-13-11-r109-S1.PDF]

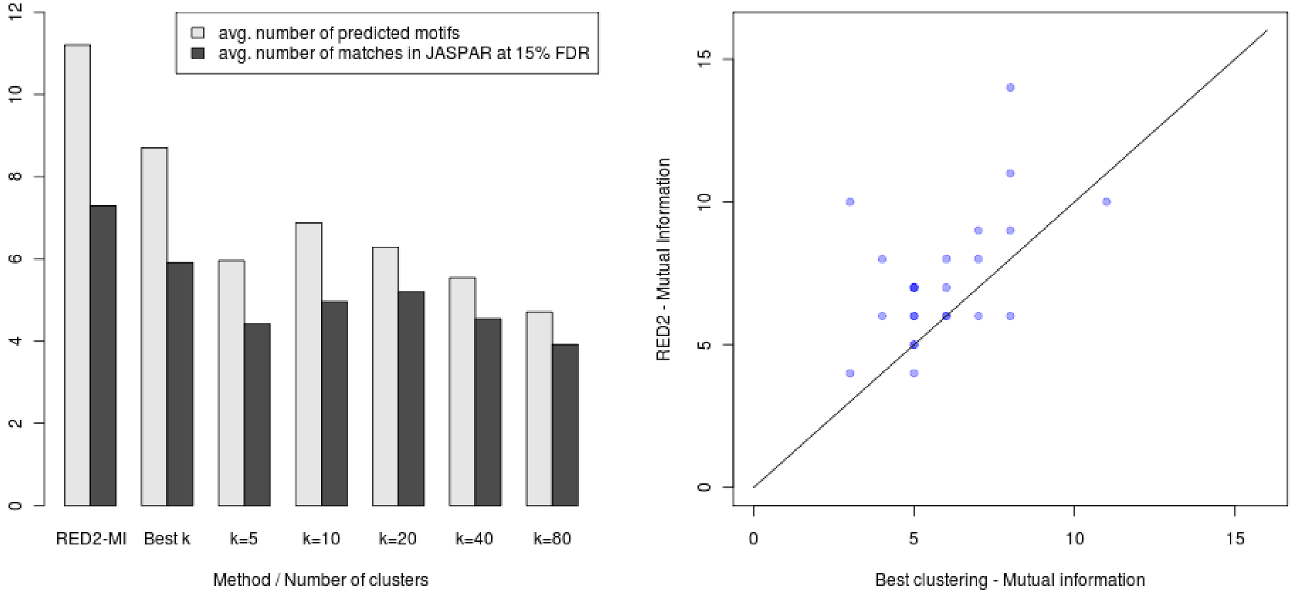

Figure 1 — Results of the mutual-information scoring functions on the JASPAR database. See Figure 8 in the main article for explanations.

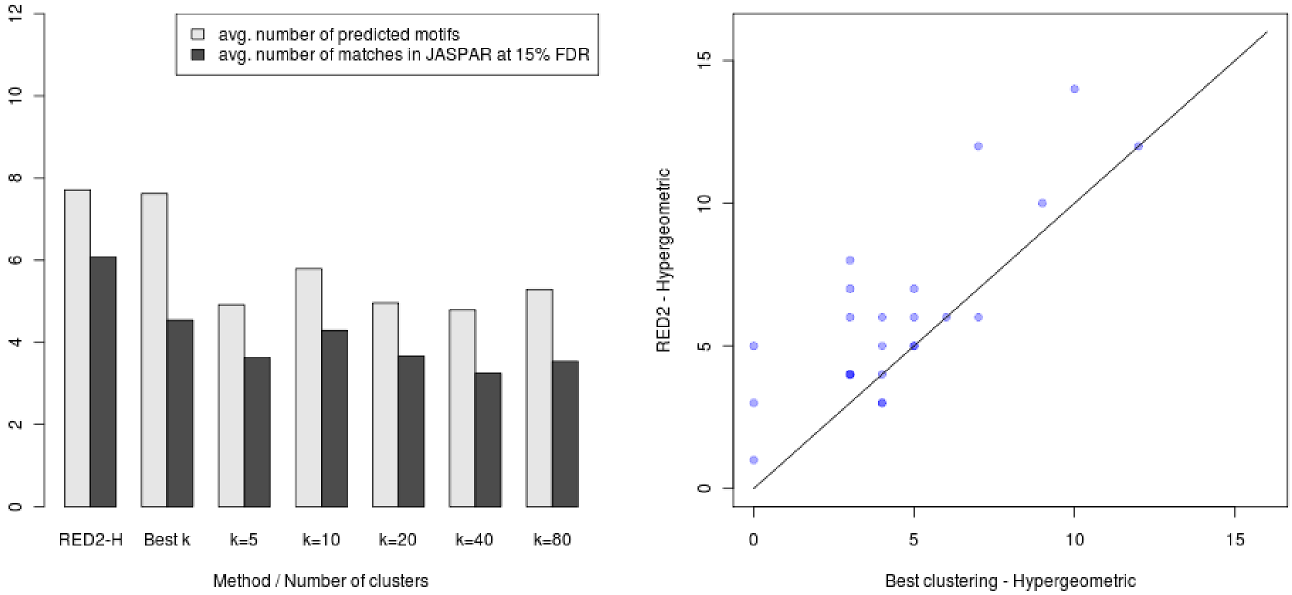

Figure 2 — Results of the hypergeometric scoring functions on the JASPAR database. See Figure 9 in the main article for explanations.

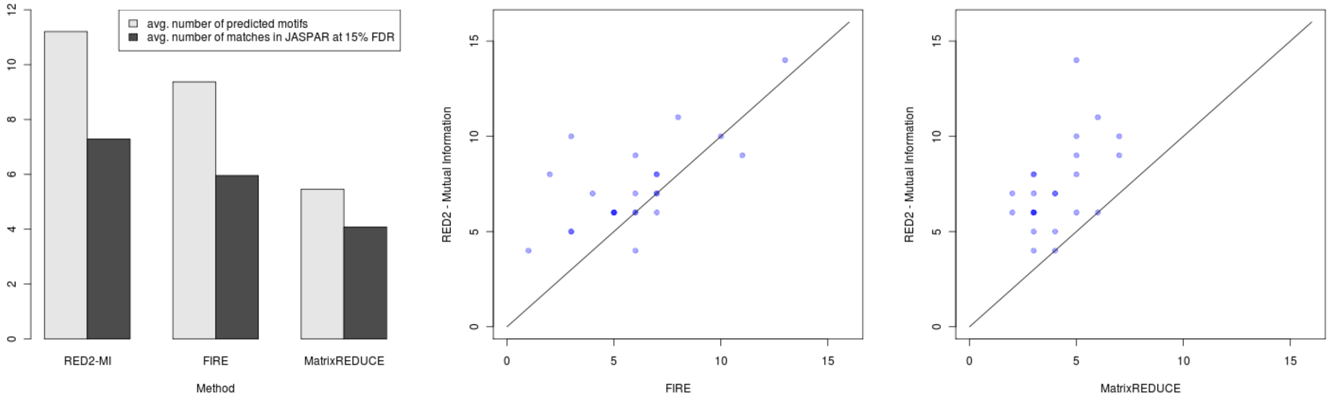

Figure 3 — Comparison between RED^2, FIRE and MatrixREDUCE on the JASPAR database. See Figure 10 in the main article for explanations.

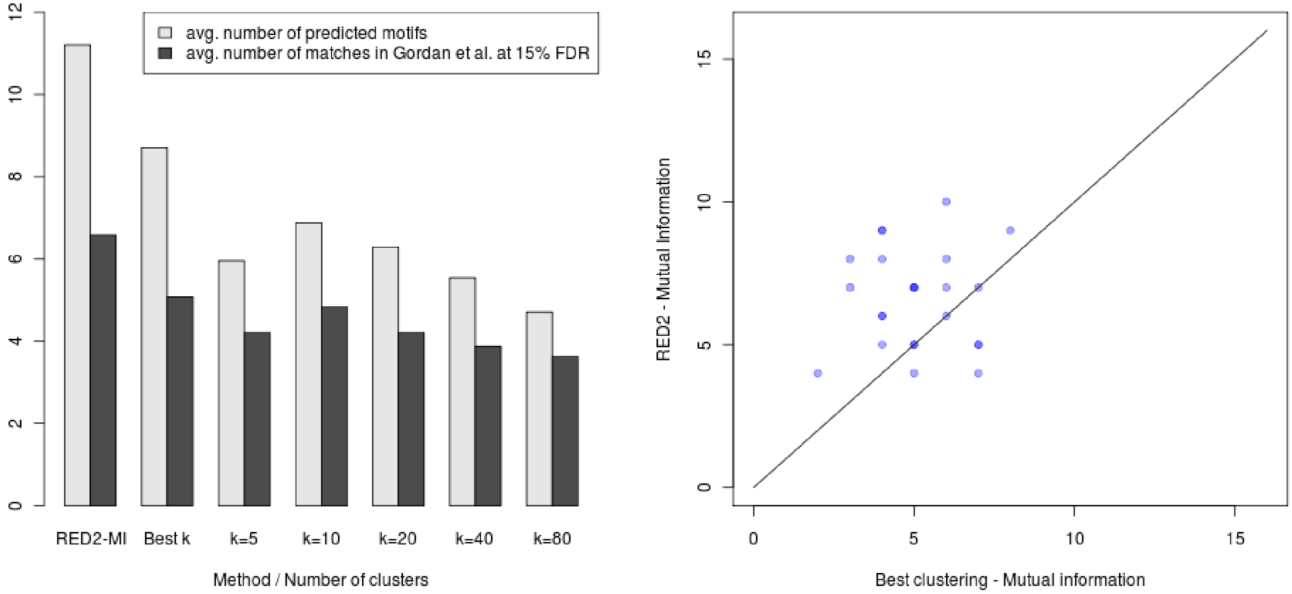

Figure 4 — Results of the mutual-information scoring functions on the Gordân et al. database. See Figure 8 in the main article for explanations.

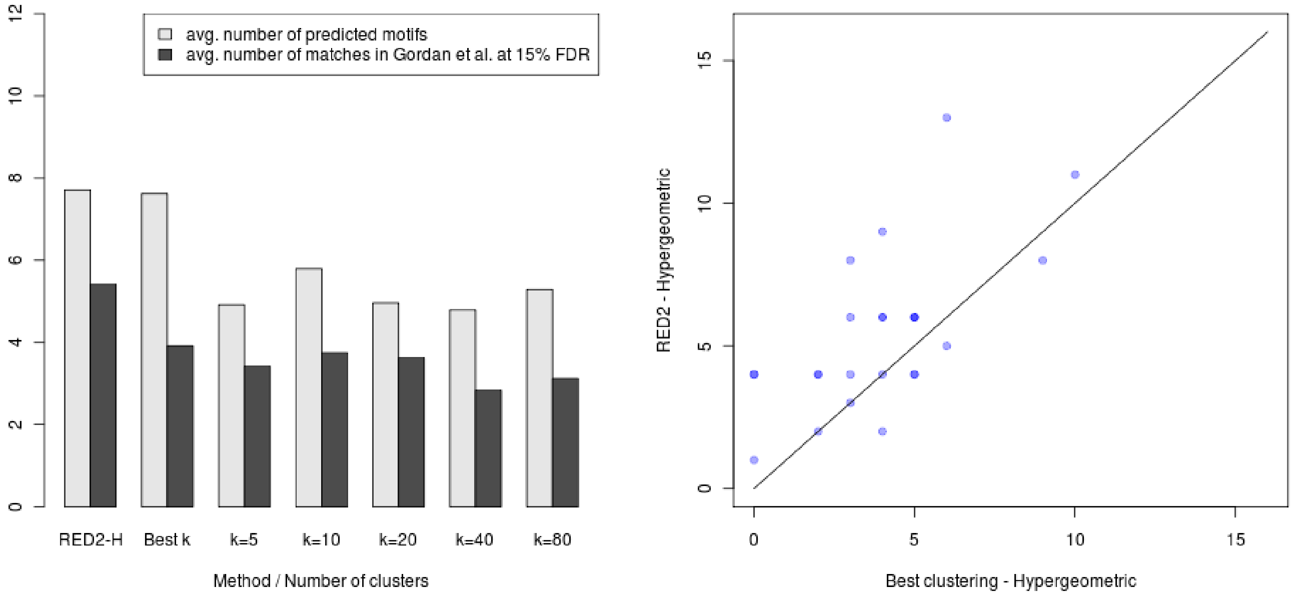

Figure 5 — Results of the hypergeometric scoring functions on the Gordân et al. database. See Figure 9 in the main article for explanations.

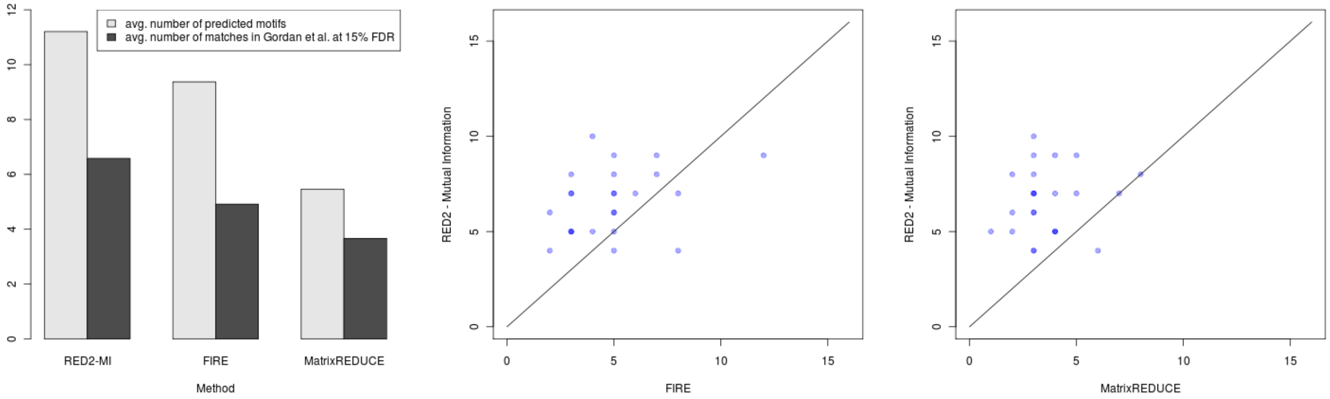

Figure 6 — Comparison between RED^2, FIRE and MatrixREDUCE on the Gordân et al. database. See Figure 10 in the main article for explanations.
