# Supplementary material for: Computational discovery of regulatory elements in a continuous expression space
Source: Genome Biol. 2012 Nov 27;13(11):R109. doi: 10.1186/gb-2012-13-11-r109 (PMC4053739; doi:10.1186/gb-2012-13-11-r109)
Supplement: Additional file 2 — Results of RED2 with mutual information criterion on the S. cerevisiae upstream regions with the Gasch et al. stress compendium. The set of motifs inferred by RED2 on the Gasch et al. complete compendium. Provided for each motif are the logo of the motif, the number of genes that have the motif in their promoter region, the expression profiles (heatmap) associated with the motif, a histogram of the motif occurrence positions, the existence/absence of any strand bias, and the name of the ScerTF motif that best matches the inferred motif (if any at 15% FDR). [file gb-2012-13-11-r109-S2.PDF]

RED2 (mutual information) on Yeast stress compendium (Gasch et al.)								
id	logo	score	#genes	expression	distances	strand	match	GO terms
#1	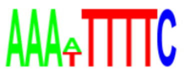	0.062	721	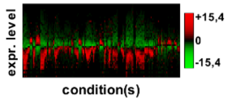	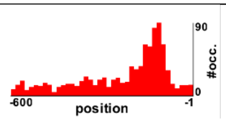		zhu_SFP1<br>P ≤3.91e-03	GO:0005730<br>nucleolus<br>P ≤4.41e-54
#2	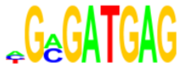	0.061	425	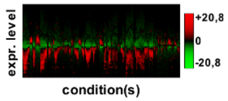	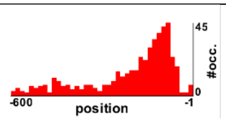		zhu_TOD6<br>P ≤1.17e-02	GO:0005730<br>nucleolus<br>P ≤1.78e-70
#3	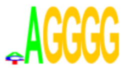	0.044	1894	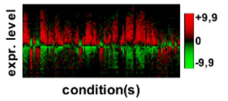	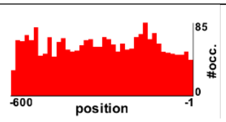		badis_GIS1<br>P ≤1.56e-02	GO:0006006<br>glucose metabolic process<br>P ≤2.50e-03
#4	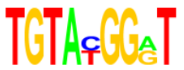	0.019	179	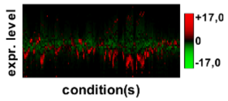	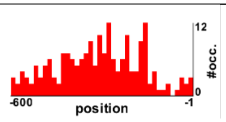		morozov_RAP1<br>P ≤3.91e-03	GO:0022626<br>cytosolic ribosome<br>P ≤1.74e-46
#5	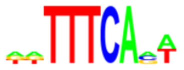	0.018	3385	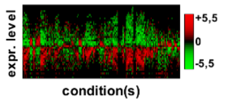	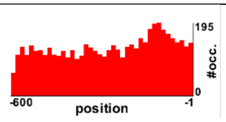		zhu_STB3<br>P ≤4.60e-02	GO:0030529<br>ribonucleoprotein complex<br>P ≤4.45e-07
#6	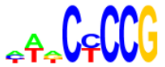	0.017	1300	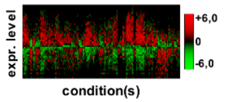	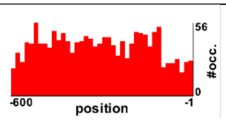		badis_YPR196W<br>P ≤3.91e-03	GO:0055114<br>oxidation-reduction process<br>P ≤2.14e-05
#7	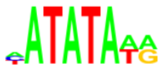	0.013	3295	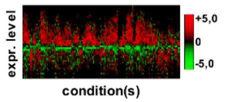	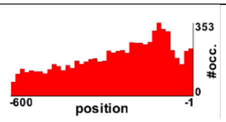	→<br>2.08e-04	pachkov_SPT15<br>P ≤1.56e-02	GO:0051187<br>cofactor catabolic process<br>P ≤2.92e-03
#8	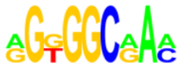	0.013	384	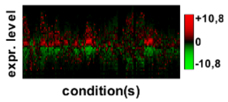	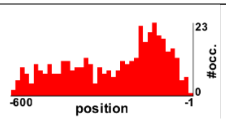		spivak_RPN4<br>P ≤3.91e-03	GO:0000502<br>proteasome complex<br>P ≤9.94e-35

#9	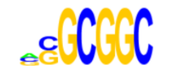	0.012	697	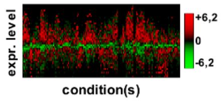	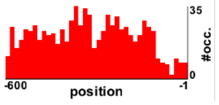		pachkov_UME6 P ≤5.71e-02	GO:0005829 cytosol P ≤4.03e-02
#10	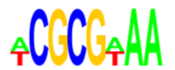	0.012	328	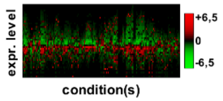	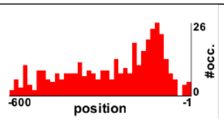		harbison_STB1 P ≤7.81e-03	GO:0005694 chromosome P ≤7.72e-20
#11	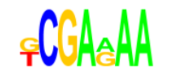	0.011	2016	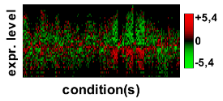	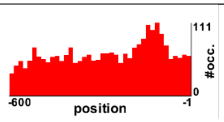			
#12	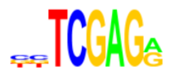	0.010	1087	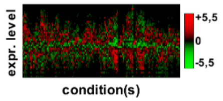	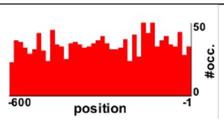		foat_XBP1 P ≤3.91e-03	GO:0030312 external encapsulating structure P ≤4.14e-03
#13	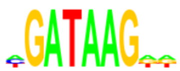	0.009	892	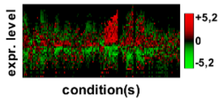	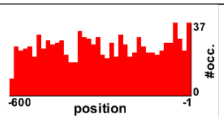		foat_DAL81 P ≤1.17e-02	GO:0043605 cellular amide catabolic process P ≤6.50e-04
#14	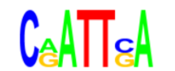	0.009	1738	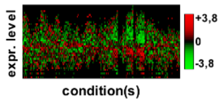	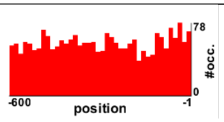			
#15	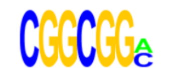	0.008	318	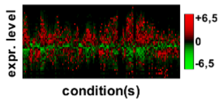	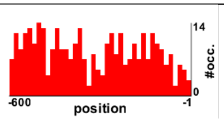		macisaac_URC2 P ≤1.94e-02	GO:0007129 synapsis P ≤1.35e-02
#16	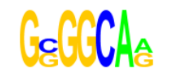	0.008	788	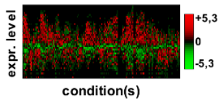	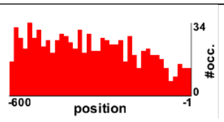			GO:0015980 energy derivation by oxidation of org... P ≤6.93e-03
#17	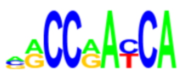	0.008	380	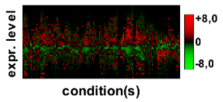	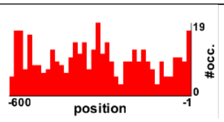		spivak_HAP3 P ≤3.91e-03	GO:0015986 ATP synthesis coupled proton transport P ≤3.46e-14

#18	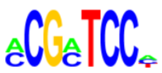	0.007	400	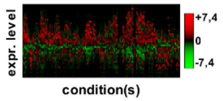	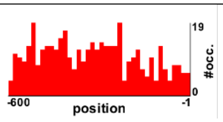			
#19	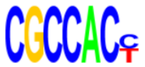	0.007	335	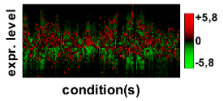	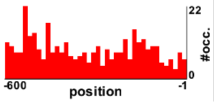			
