# Supplementary material for: Computational discovery of regulatory elements in a continuous expression space
Source: Genome Biol. 2012 Nov 27;13(11):R109. doi: 10.1186/gb-2012-13-11-r109 (PMC4053739; doi:10.1186/gb-2012-13-11-r109)
Supplement: Additional file 3 — Results of RED2 (hypergeometric) on S. cerevisiae upstream regions with the Gasch et al. stress compendium. The set of motifs inferred by RED2 on the Gasch et al. complete compendium. See the description of Additional file 2 for table column definitions. [file gb-2012-13-11-r109-S3.PDF]

RED2 (hypergeometric) on Yeast stress compendium (Gasch et al.)								
id	logo	score	#genes	expression	distances	strand	match	GO terms
#1	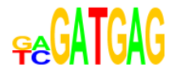	88.170	735	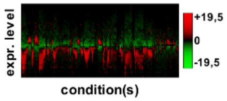	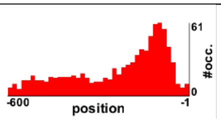		zhu_DOT6 P ≤3.91e-03	GO:0005730 nucleolus P ≤1.89e-67
#2	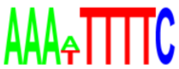	81.517	721	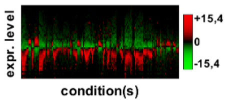	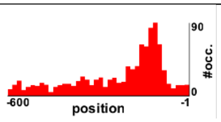		zhu_SFP1 P ≤3.91e-03	GO:0005730 nucleolus P ≤4.41e-54
#3	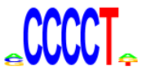	57.628	1276	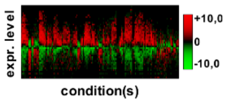	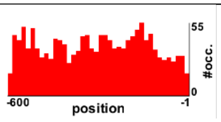		harbison_MSN2 P ≤3.91e-03	GO:0006006 glucose metabolic process P ≤1.93e-04
#4	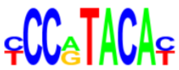	43.440	224	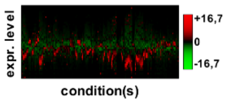	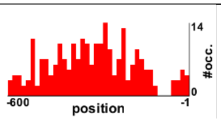	→ 7.74e-03	morozov_RAP1 P ≤3.91e-03	GO:0022626 cytosolic ribosome P ≤1.09e-47
#5	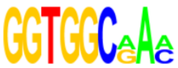	29.803	158	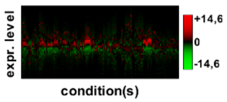	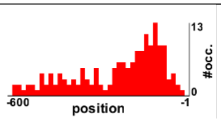		spivak_RPN4 P ≤3.91e-03	GO:0000502 proteasome complex P ≤1.03e-32
#6	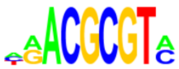	20.120	178	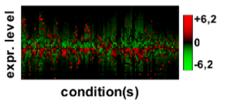	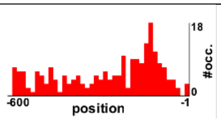		spivak_MBP1 P ≤7.18e-02	GO:0006259 DNA metabolic process P ≤6.86e-14
#7	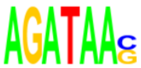	18.783	1032	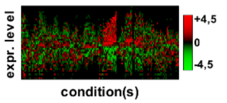	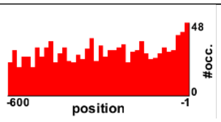		foat_DAL81 P ≤3.09e-02	
#8	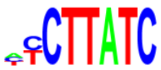	18.505	821	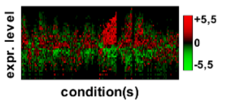	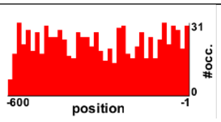		harbison_GLN3 P ≤3.91e-03	GO:0043605 cellular amide catabolic process P ≤1.60e-02

#9	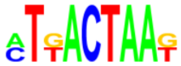	17.997	304	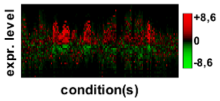	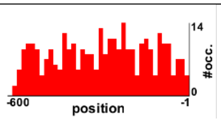		spivak_YAP7<br>P ≤3.91e-03	GO:0016209<br>antioxidant activity<br>P ≤2.46e-05
#10	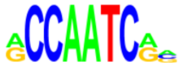	17.939	240	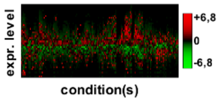	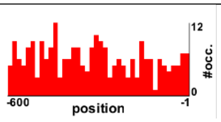		pachkov_HAP5<br>P ≤3.91e-03	GO:0015986<br>ATP synthesis coupled proton transport<br>P ≤1.18e-09
#11	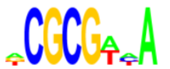	17.180	785	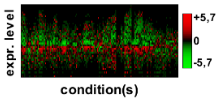	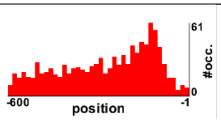		harbison_STB1<br>P ≤5.71e-02	GO:0005694<br>chromosome<br>P ≤8.68e-22
#12	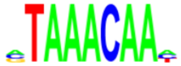	14.112	769	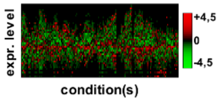	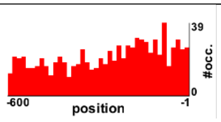	→<br>7.33e-03	badis_HCM1<br>P ≤9.34e-02	GO:0005856<br>cytoskeleton<br>P ≤1.76e-07
#13	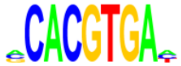	11.291	191	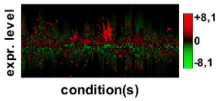	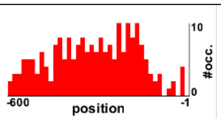		macisaac_CBF1<br>P ≤3.91e-03	GO:0000096<br>sulfur amino acid metabolic process<br>P ≤4.97e-12
#14	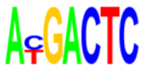	10.175	469	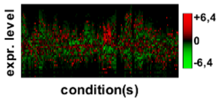	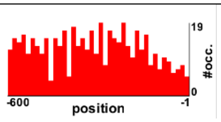		macisaac_RTG3<br>P ≤8.62e-02	GO:0008652<br>cellular amino acid biosynthetic process<br>P ≤2.74e-10
