# Supplementary material for: Computational discovery of regulatory elements in a continuous expression space
Source: Genome Biol. 2012 Nov 27;13(11):R109. doi: 10.1186/gb-2012-13-11-r109 (PMC4053739; doi:10.1186/gb-2012-13-11-r109)
Supplement: Additional file 4 — Results of RED2 (mutual information) on S. cerevisiae upstream regions with the Spellman et al. cell-cycle dataset. The set of motifs inferred by RED2 on the Spellman et al. dataset. See the description of Additional file 2 for table column definitions. [file gb-2012-13-11-r109-S4.PDF]

RED2 (mutual information) on Yeast cell cycle (Spellman et al.)								
id	logo	score	#genes	expression	distances	strand	match	GO terms
#1	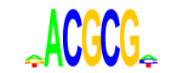	0.055	1096	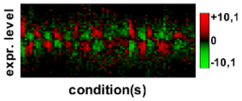	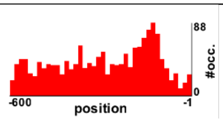		spivak_MBP1<br>P ≤3.09e-02	GO:0006259<br>DNA metabolic process<br>P ≤1.00e-14
#2	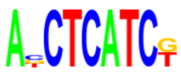	0.043	451	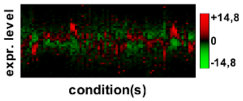	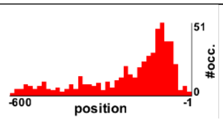		zhu_TOD6<br>P ≤3.91e-03	GO:0005730<br>nucleolus<br>P ≤1.54e-73
#3	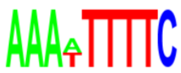	0.041	721	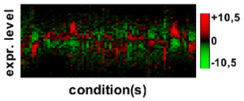	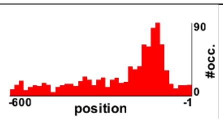		zhu_SFP1<br>P ≤3.91e-03	GO:0005730<br>nucleolus<br>P ≤6.40e-54
#4	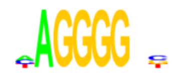	0.020	1286	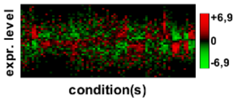	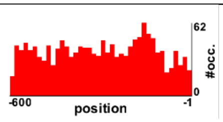		harbison_MSN2<br>P ≤1.94e-02	GO:0006006<br>glucose metabolic process<br>P ≤2.95e-03
#5	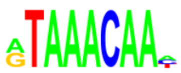	0.012	639	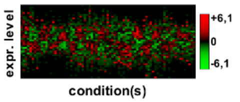	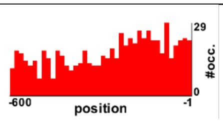			GO:0005856<br>cytoskeleton<br>P ≤4.37e-08
#6	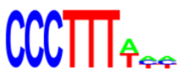	0.011	1084	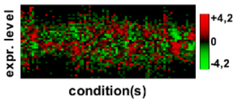	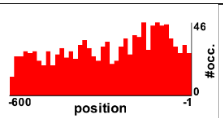			GO:0030258<br>lipid modification<br>P ≤1.49e-02
#7	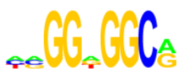	0.010	518	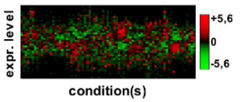	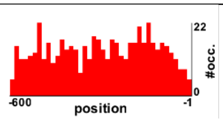			GO:0000502<br>proteasome complex<br>P ≤1.54e-15
#8	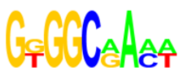	0.010	362	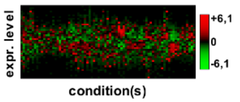	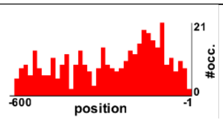		spivak_RPN4<br>P ≤2.32e-02	GO:0000502<br>proteasome complex<br>P ≤1.01e-28

#9	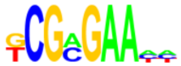	0.009	776	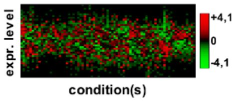	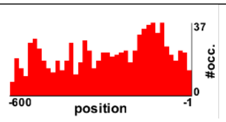			
#10	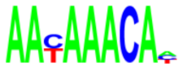	0.009	636	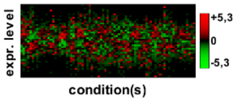	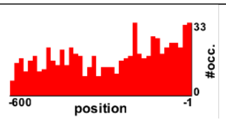	→ 1.74e-07		GO:0007059 chromosome segregation P ≤1.37e-02
#11	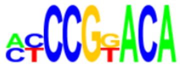	0.009	131	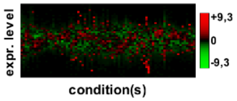	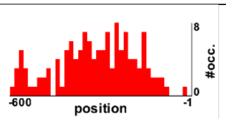		morozov_RAP1 P ≤2.32e-02	GO:0022626 cytosolic ribosome P ≤1.05e-25
#12	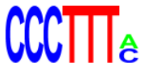	0.009	1011	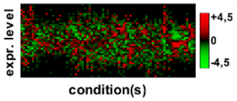	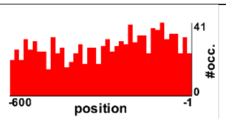			
#13	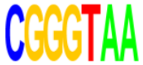	0.009	641	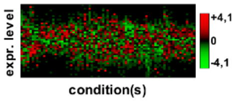	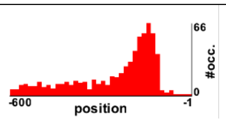		pachkov_YDR026C P ≤3.91e-03	GO:0032991 macromolecular complex P ≤3.13e-06
#14	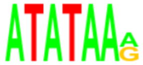	0.007	2642	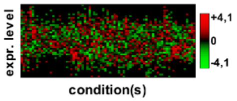	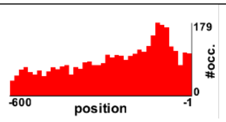	→ 4.72e-07	pachkov_SPT15 P ≤7.81e-03	GO:0055114 oxidation-reduction process P ≤4.81e-04
#15	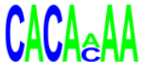	0.007	1203	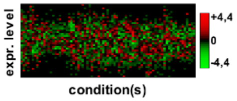	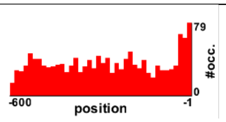		zhu_NDT80 P ≤2.71e-02	GO:0030154 cell differentiation P ≤4.55e-04
