# Supplementary material for: Computational discovery of regulatory elements in a continuous expression space
Source: Genome Biol. 2012 Nov 27;13(11):R109. doi: 10.1186/gb-2012-13-11-r109 (PMC4053739; doi:10.1186/gb-2012-13-11-r109)
Supplement: Additional file 5 — Results of RED2 (hypergeometric) on S. cerevisiae upstream regions with the Spellman et al. cell-cycle dataset. The set of motifs inferred by RED2 on the Spellman et al. dataset. See the description of Additional file 2 for table column definitions. [file gb-2012-13-11-r109-S5.PDF]

RED2 (hypergeometric) on Yeast cell cycle (Spellman et al.)								
id	logo	score	#genes	expression	distances	strand	match	GO terms
#1	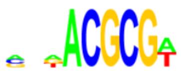	74.388	655	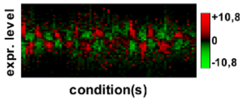	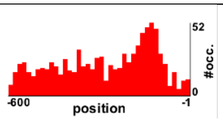		spivak_MBP1 P $\leq$6.81e-02	GO:0006259 DNA metabolic process P $\leq$2.13e-18
#2	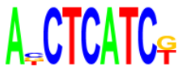	59.871	451	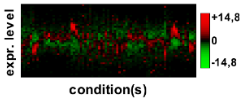	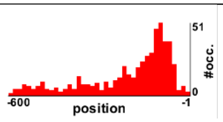		zhu_TOD6 P $\leq$3.91e-03	GO:0005730 nucleolus P $\leq$1.54e-73
#3	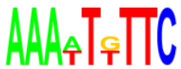	45.018	876	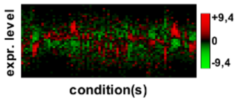	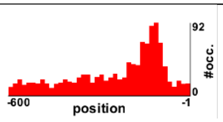		zhu_SFP1 P $\leq$3.91e-03	GO:0005730 nucleolus P $\leq$8.89e-49
#4	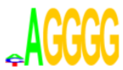	26.700	1883	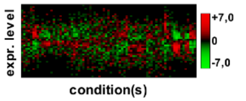	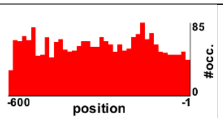		badis_GIS1 P $\leq$1.56e-02	GO:0006006 glucose metabolic process P $\leq$2.46e-03
#5	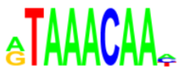	25.468	639	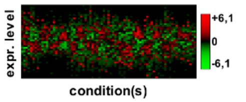	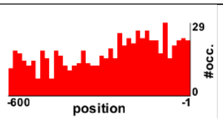			GO:0005856 cytoskeleton P $\leq$4.37e-08
#6	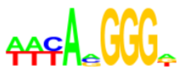	23.052	1222	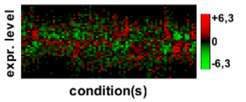	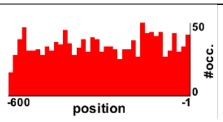			GO:0044262 cellular carbohydrate metabolic process P $\leq$7.82e-05
#7	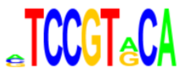	19.854	121	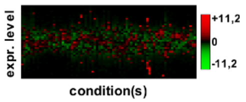	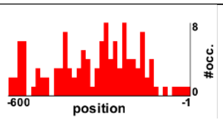			GO:0022626 cytosolic ribosome P $\leq$3.65e-30
#8	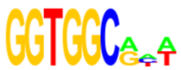	17.888	252	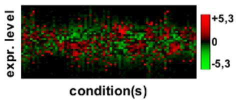	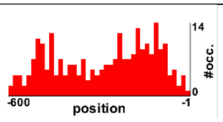		spivak_RPN4 P $\leq$3.91e-03	GO:0000502 proteasome complex P $\leq$4.17e-28

#9	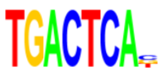	17.388	187	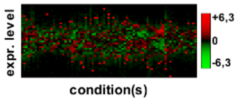	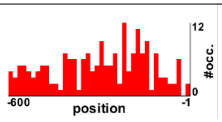		spivak_GCN4<br>P ≤3.91e-03	GO:0008652<br>cellular amino acid biosynthetic process<br>P ≤8.73e-25
#10	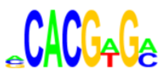	13.991	512	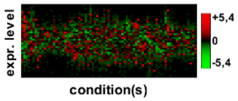	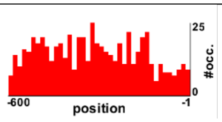		morozov_PHO4<br>P ≤6.08e-02	GO:0000097<br>sulfur amino acid biosynthetic process<br>P ≤2.22e-06
#11	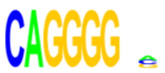	12.615	366	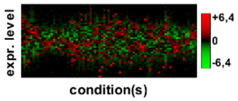	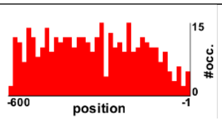		badis_REI1<br>P ≤7.54e-02	GO:0005984<br>disaccharide metabolic process<br>P ≤3.93e-05
#12	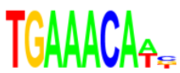	12.569	383	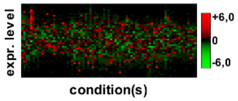	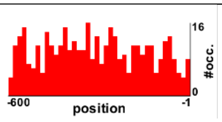		badis_STE12<br>P ≤7.18e-02	GO:0019236<br>response to pheromone<br>P ≤3.99e-05
#13	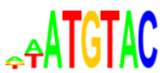	10.385	911	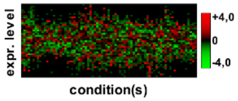	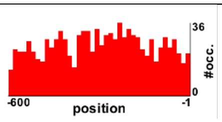			GO:0002181<br>cytoplasmic translation<br>P ≤1.61e-06
#14	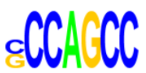	9.578	267	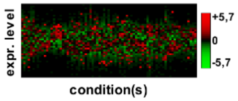	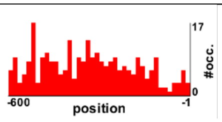			
