# Supplementary material for: Computational discovery of regulatory elements in a continuous expression space
Source: Genome Biol. 2012 Nov 27;13(11):R109. doi: 10.1186/gb-2012-13-11-r109 (PMC4053739; doi:10.1186/gb-2012-13-11-r109)
Supplement: Additional file 6 — Results of FIRE on S. cerevisiae upstream regions with the Spellman et al. cell-cycle dataset. The set of motifs inferred by FIRE on the Spellman et al. dataset. See the description of Additional file 2 for table column definitions. [file gb-2012-13-11-r109-S6.PDF]

FIRE on Yeast cell cycle (Spellman et al.)								
id	logo	score	#genes	expression	distances	strand	match	GO terms
#1	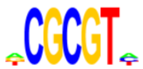	NA	1096	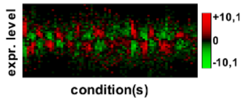	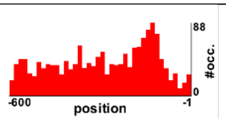		spivak_MBP1<br>P ≤3.09e-02	GO:0006259<br>DNA metabolic process<br>P ≤1.00e-14
#2	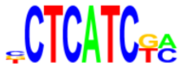	NA	621	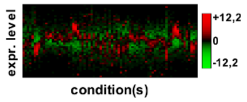	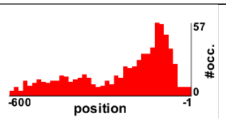		zhu_DOT6<br>P ≤3.91e-03	GO:0005730<br>nucleolus<br>P ≤3.32e-68
#3	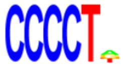	NA	1883	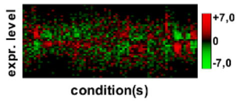	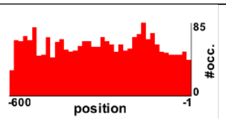		badis_GIS1<br>P ≤1.56e-02	GO:0006006<br>glucose metabolic process<br>P ≤2.46e-03
#4	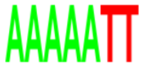	NA	2382	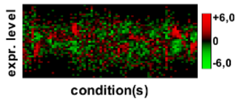	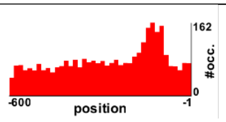		zhu_SFP1<br>P ≤4.22e-02	GO:0042254<br>ribosome biogenesis<br>P ≤5.04e-25
#5	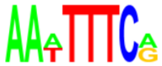	NA	1512	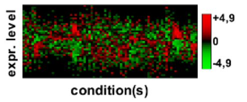	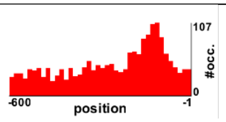			GO:0042254<br>ribosome biogenesis<br>P ≤1.38e-12
#6	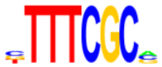	NA	774	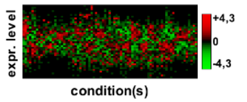	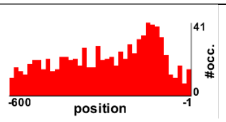			
#7	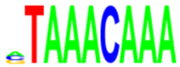	NA	425	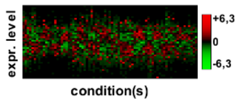	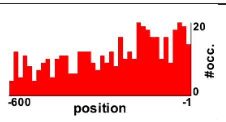	→<br>1.18e-03		GO:0005875<br>microtubule associated complex<br>P ≤2.12e-04
#8	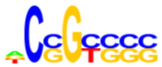	NA	1836	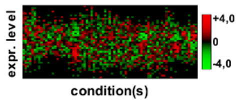	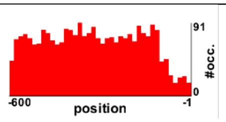			GO:0055114<br>oxidation-reduction process<br>P ≤1.95e-02

#9	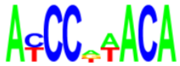	NA	379	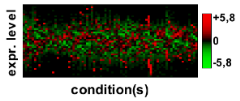	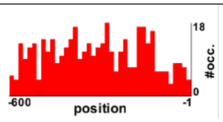		morozov_RAP1<br>P ≤3.91e-03	GO:0022626<br>cytosolic ribosome<br>P ≤2.15e-41
#10	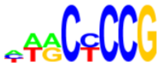	NA	781	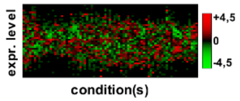	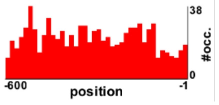			GO:0055114<br>oxidation-reduction process<br>P ≤8.12e-03
#11	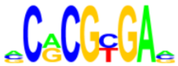	NA	302	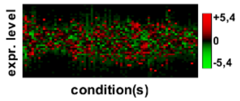	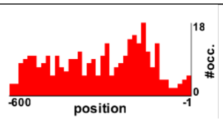			GO:0044271<br>cellular nitrogen compound biosynthet...<br>P ≤2.87e-02
#12	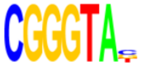	NA	411	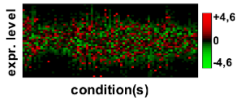	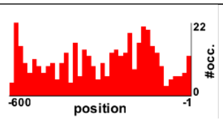			
