# Supplementary material for: Computational discovery of regulatory elements in a continuous expression space
Source: Genome Biol. 2012 Nov 27;13(11):R109. doi: 10.1186/gb-2012-13-11-r109 (PMC4053739; doi:10.1186/gb-2012-13-11-r109)
Supplement: Additional file 7 — Results of MatrixREDUCE on S. cerevisiae upstream regions with the Spellman et al. cell-cycle dataset. The set of motifs inferred by MatrixREDUCE on the Spellman et al. dataset. See the description of Additional file 2 for table column definitions. [file gb-2012-13-11-r109-S7.PDF]

MatrixREDUCE on Yeast cell cycle (Spellman et al.)								
id	logo	score	#genes	expression	distances	strand	match	GO terms
#1	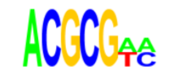	NA	587	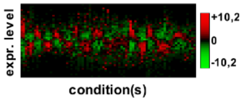	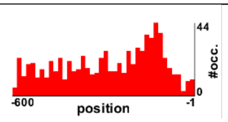		harbison_STB1<br>P ≤1.04e-01	GO:0006259<br>DNA metabolic process<br>P ≤3.08e-21
#2	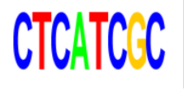	NA	298	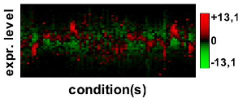	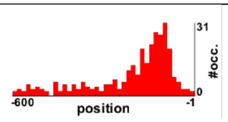		zhu_TOD6<br>P ≤3.91e-03	GO:0005730<br>nucleolus<br>P ≤3.36e-51
#3	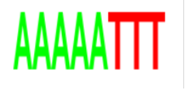	NA	1103	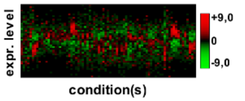	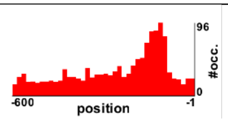		zhu_SFP1<br>P ≤3.91e-03	GO:0042254<br>ribosome biogenesis<br>P ≤8.28e-38
#4	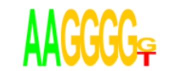	NA	473	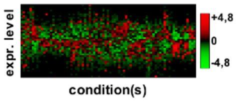	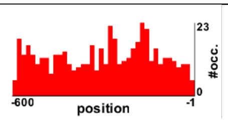			GO:0006112<br>energy reserve metabolic process<br>P ≤1.17e-03
#5	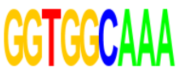	NA	98	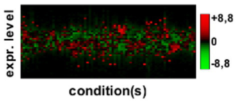	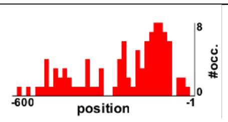		spivak_RPN4<br>P ≤3.91e-03	GO:0000502<br>proteasome complex<br>P ≤1.50e-33
#6	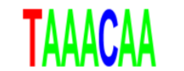	NA	1198	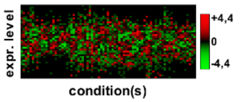	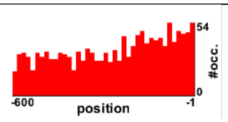	→<br>9.71e-03	foat_FKH1<br>P ≤2.71e-02	GO:0005856<br>cytoskeleton<br>P ≤2.43e-04
#7	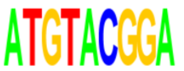	NA	40	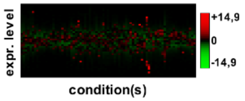	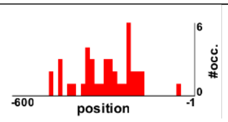		morozov_RAP1<br>P ≤6.81e-02	GO:0022626<br>cytosolic ribosome<br>P ≤3.08e-17
#8	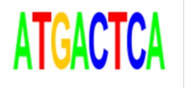	NA	83	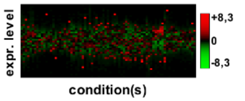	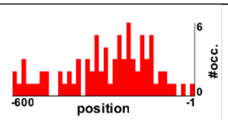		macisaac_RTG3<br>P ≤7.81e-03	GO:0008652<br>cellular amino acid biosynthetic process<br>P ≤5.90e-10

#9	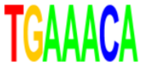	NA	849	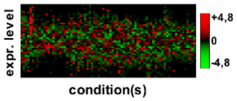	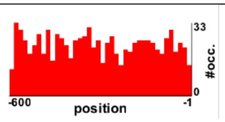		badis_STE12<br>P $\leq$3.91e-03	GO:0019236<br>response to pheromone<br>P $\leq$3.71e-04
