# Supplementary material for: Computational discovery of regulatory elements in a continuous expression space
Source: Genome Biol. 2012 Nov 27;13(11):R109. doi: 10.1186/gb-2012-13-11-r109 (PMC4053739; doi:10.1186/gb-2012-13-11-r109)
Supplement: Additional file 8 — ScerTF motifs recovered by RED2, FIRE and MatrixREDUCE on the 24 S. cerevisiae datasets. For each method, this table provides the number of datasets in which each motif was recovered (at 15% FDR). For example, REDUCE correctly predicts AFT1 (first line) in one dataset. The last two lines give the total number of correct predictions (Total) and the number of distinct motifs recovered (Coverage). For example, for the 24 datasets, RED2 makes 177 correct predictions, which cover 43 distinct motifs in ScerTF. [file gb-2012-13-11-r109-S8.PDF]

Motif	RED2-MI	RED2-H	FIRE	MatrixREDUCE
AFT1	0	0	0	1
AFT2	0	0	0	1
ARG81	1	1	0	0
ASG1	0	0	1	0
ASH1	0	0	1	0
BAS1	0	0	2	0
CAD1	0	0	0	2
CBF1	0	3	1	0
CEP3	1	0	0	0
CIN5	0	0	0	1
DAL81	3	2	0	0
DOT6	0	8	4	0
FKH1	0	0	0	1
FKH2	0	1	0	0
GAT1	1	2	0	0
GCN4	1	3	1	1
GIS1	6	1	6	0
GLN3	1	1	1	0
GZF3	0	0	1	0
HAC1	2	0	0	0
HAP3	4	4	4	0
HAP4	0	0	1	0
HAP5	0	1	0	0
HCM1	0	1	0	0
HSF1	0	0	0	2
IME1	1	0	0	0
INO4	2	0	0	0
MBP1	6	5	1	0
MCM1	0	0	1	0
MET31	1	0	1	0
MET32	1	2	2	3
MIG2	2	0	2	0
MIG3	1	2	0	0
MSN2	8	13	8	2
NDT80	2	0	0	0
NHP10	1	0	0	0
NHP6A	2	1	1	0
NHP6B	1	1	0	0
PHO4	0	1	0	0
PIP2	0	0	1	0
RAP1	22	18	19	13
REB1	3	0	0	0
REI1	0	2	1	6
RGM1	1	0	0	0
RIM101	1	0	0	0
RPH1	0	2	0	0
RPN4	10	11	5	4
RSF2	1	0	0	0
RTG3	3	2	1	2
SFP1	23	23	5	17

SKO1	0	1	1	0
SNF1	1	0	0	0
SNT2	0	1	0	0
SPT15	12	5	8	0
STB1	9	2	5	1
STB2	2	0	0	0
STB3	5	0	3	8
STE12	0	1	0	1
STP2	0	1	0	1
SUT1	0	0	1	0
SWI4	5	1	3	0
TOD6	24	15	17	20
TYE7	1	0	0	0
UME1	0	0	1	0
UME6	2	0	1	0
URC2	2	0	0	0
XBP1	2	1	2	0
YAP1	0	0	0	3
YAP7	2	2	3	0
YDR026C	1	0	0	0
YER130C	4	3	5	0
YLR278C	0	1	1	0
YNR063W	0	0	1	0
YPR196W	1	0	2	0
YRR1	1	0	0	0
Total	186	145	125	90
Coverage	45	37	39	20
