# Supplementary material for: Computational discovery of regulatory elements in a continuous expression space
Source: Genome Biol. 2012 Nov 27;13(11):R109. doi: 10.1186/gb-2012-13-11-r109 (PMC4053739; doi:10.1186/gb-2012-13-11-r109)
Supplement: Additional file 9 — Results of RED2 (mutual information) on P. falciparum upstream regions with the Bozdech et al. dataset (erythrocytic cycle). The set of motifs inferred by RED2 on the upstream regions of P. falciparum genes using the Bozdech et al. dataset [11]. See the description of Additional file 2 for a description of the different columns. [file gb-2012-13-11-r109-S9.PDF]

RED2 (mutual information) on P.falciparum intraerythrocytic cycle (Bozdech et al.)								
id	logo	score	#genes	expression	distances	strand	match	GO terms
#1	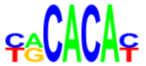	0.175	2127	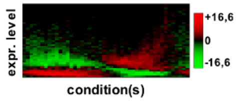	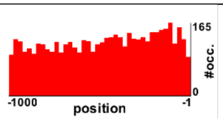	← 1.26e-04	MAL8P1.153 P ≤9.20e-04	GO:0020011 apicoplast P ≤1.91e-04
#2	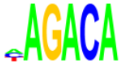	0.092	1263	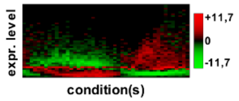	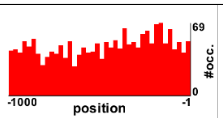			GO:0004672 protein kinase activity P ≤4.14e-04
#3	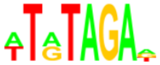	0.086	2378	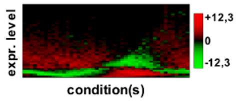	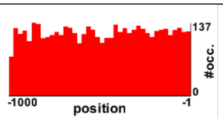	→ 5.01e-09	PFL1900w_D1 P ≤1.38e-03	
#4	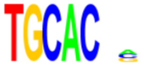	0.049	667	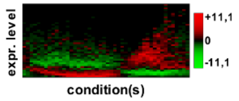	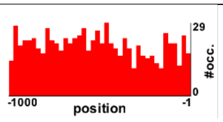		PF10_0075_D3 P ≤1.10e-02	GO:0008092 cytoskeletal protein binding P ≤7.78e-02
#5	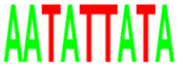	0.026	2263	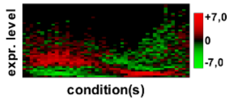	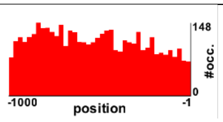			GO:0005829 cytosol P ≤4.45e-04
#6	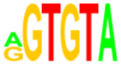	0.025	1266	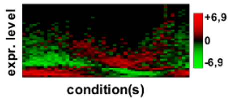	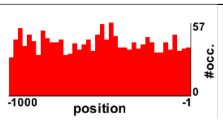			
#7	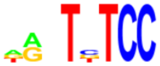	0.021	1925	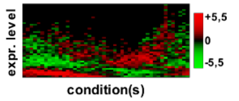	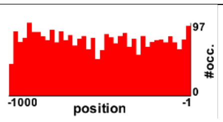	→ 4.64e-04	PF07_0126_DLD P ≤7.33e-03	
#8	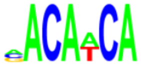	0.020	1385	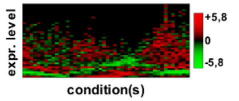	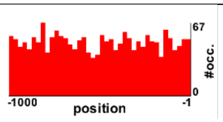	← 1.12e-11	PFE0840c_D2 P ≤2.81e-02	

#9	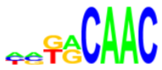	0.020	1043	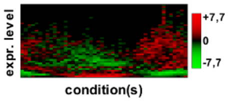	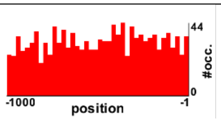	← 7.38e-03		GO:0051704 multi-organism process P ≤1.32e-02
#10	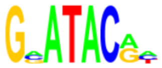	0.019	1073	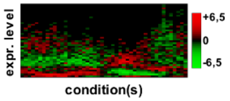	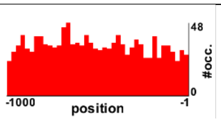		PF11_0091 P ≤9.20e-04	
#11	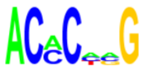	0.017	510	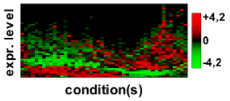	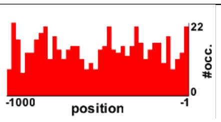		PF13_0026 P ≤5.76e-02	
#12	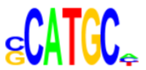	0.017	180	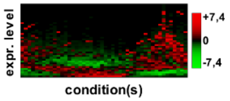	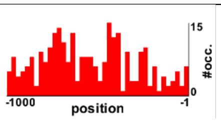			GO:0046658 anchored to plasma membrane P ≤1.27e-02
#13	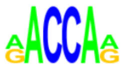	0.016	1255	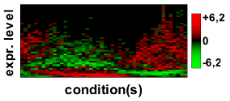	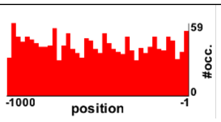			
#14	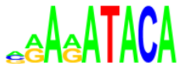	0.016	1307	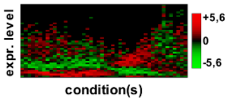	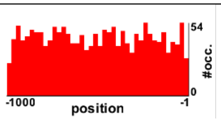	← 2.52e-05		
#15	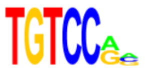	0.015	218	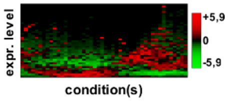	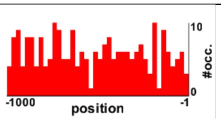			GO:0045333 cellular respiration P ≤8.60e-02
#16	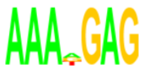	0.015	1568	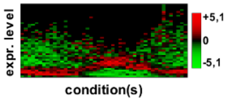	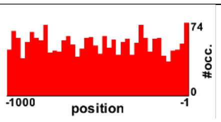			

#17	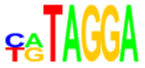	0.011	810	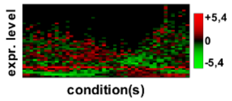	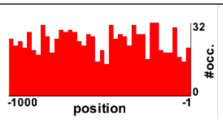			
#18	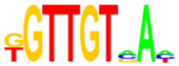	0.010	446	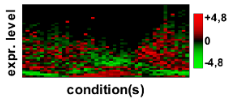	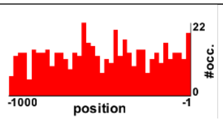		PFE0840c_D2<br>P ≤5.58e-02	GO:0006952<br>defense response<br>P ≤7.15e-05
#19	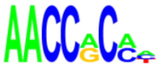	0.010	216	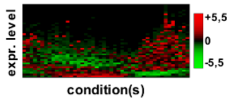	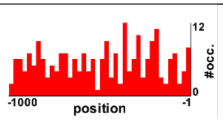			
#20	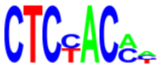	0.010	209	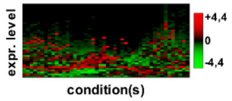	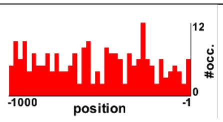			
#21	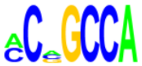	0.009	54	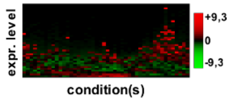	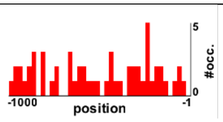		PF13_0235_D1<br>P ≤1.46e-02	
