# Supplementary material for: Computational discovery of regulatory elements in a continuous expression space
Source: Genome Biol. 2012 Nov 27;13(11):R109. doi: 10.1186/gb-2012-13-11-r109 (PMC4053739; doi:10.1186/gb-2012-13-11-r109)
Supplement: Additional file 10 — Results of RED2 (mutual information) on P. falciparum downstream regions with the Shock et al. dataset (mRNA decay). The set of motifs inferred by RED2 on the downstream regions of P. falciparum genes using the Shock et al. dataset [36]. See the description of Additional file 2 for table column definitions. [file gb-2012-13-11-r109-S10.PDF]

P.falciparum mRNA decay (Shock et al.) - 3'UTR							
id	logo	score	#genes	expression	distances	strand	match	GO terms
#1	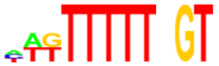	0.066	1774	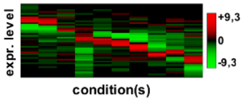	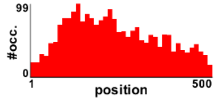	NA	Wu et al.(2008);Hall et al.(2005)	GO:0009536 plastid P $\leq$5.02e-02
#2	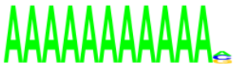	0.021	2337	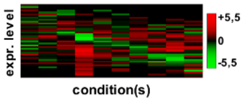	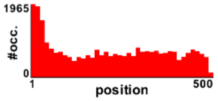	NA		
#3	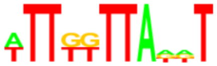	0.015	2850	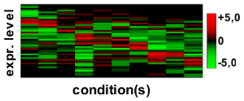	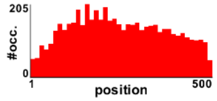	NA		
#4	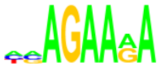	0.011	1857	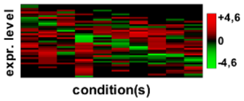	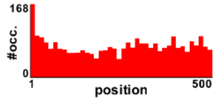	NA		
#5	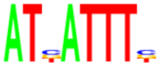	0.009	2514	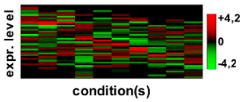	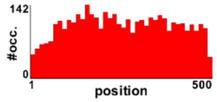	NA		
#6	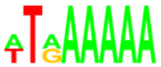	0.008	3368	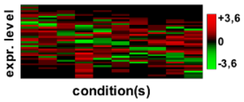	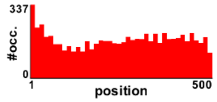	NA		
