# Supplementary material for: Computational discovery of regulatory elements in a continuous expression space
Source: Genome Biol. 2012 Nov 27;13(11):R109. doi: 10.1186/gb-2012-13-11-r109 (PMC4053739; doi:10.1186/gb-2012-13-11-r109)
Supplement: Additional file 11 — Results of FIRE on P. falciparum upstream regions with the Bozdech et al. dataset (erythrocytic cycle). The set of motifs inferred by FIRE on the upstream regions of P. falciparum genes using the Bozdech et al. dataset [11]. See the description of Additional file 2 for table column definitions. [file gb-2012-13-11-r109-S11.PDF]

FIRE on P.falciparum intraerythrocytic cycle (Bozdech et al.)

id	logo	score	#genes	expression	distances	strand	match	GO terms
#1	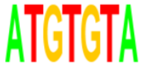	NA	1479	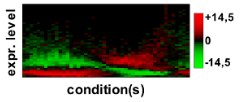	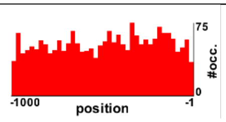			
#2	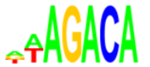	NA	1123	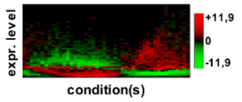	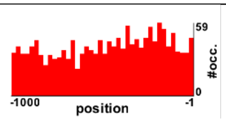			GO:0004672<br>protein kinase activity<br>P $\leq$6.27e-05
#3	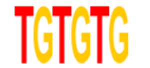	NA	1124	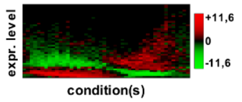	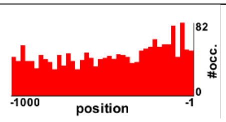	→<br>2.98e-06	PF14_0533<br>P $\leq$4.60e-04	GO:0006259<br>DNA metabolic process<br>P $\leq$1.69e-03
#4	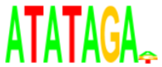	NA	1636	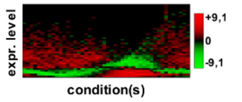	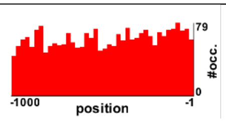	→<br>2.82e-04		
#5	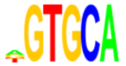	NA	1040	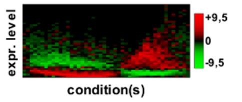	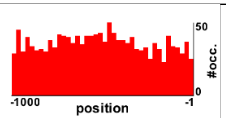		PF10_0075_D3<br>P $\leq$1.37e-02	GO:0016255<br>attachment of GPI anchor to protein<br>P $\leq$1.29e-02
#6	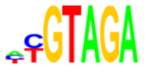	NA	1108	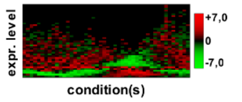	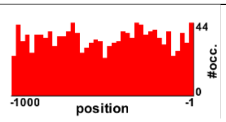	→<br>1.71e-07	PFL1900w_D1<br>P $\leq$1.01e-02	
#7	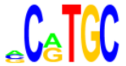	NA	667	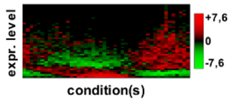	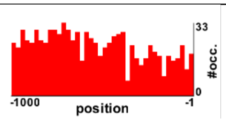			
#8	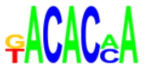	NA	1130	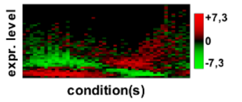	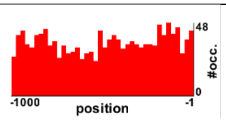	←<br>7.16e-08	PFL1085w<br>P $\leq$5.51e-03	

#9	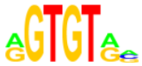	NA	978	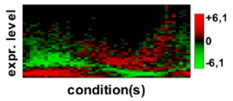	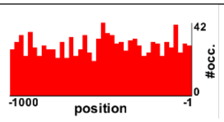			
#10	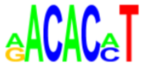	NA	1111	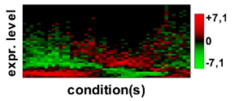	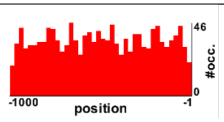		PFD0985w_D2<br>P $\leq$4.13e-03	
#11	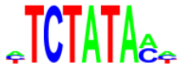	NA	1109	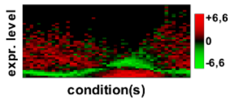	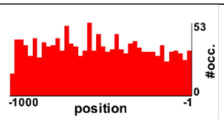			
#12	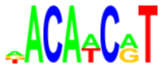	NA	1099	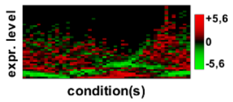	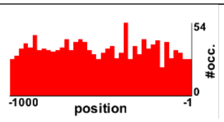			
#13	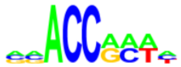	NA	1056	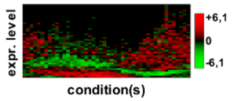	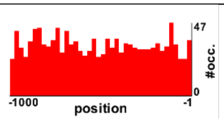	←<br>8.70e-03		GO:0033643<br>host cell part<br>P $\leq$5.60e-03
#14	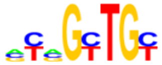	NA	590	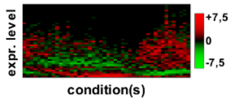	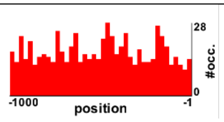			GO:0006952<br>defense response<br>P $\leq$1.85e-02
#15	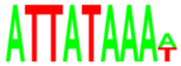	NA	2389	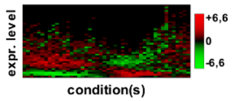	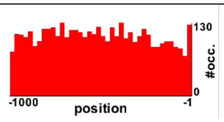	←<br>1.46e-06		
#16	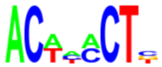	NA	896	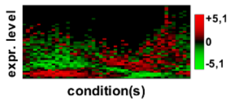	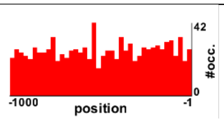			

#17	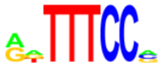	NA	991	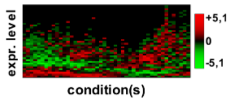	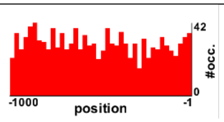	→ 2.75e-07	PF07_0126_DLD<br>P ≤9.20e-04	
#18	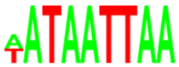	NA	1804	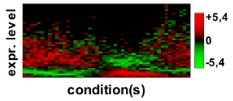	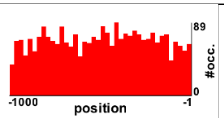			
#19	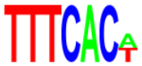	NA	969	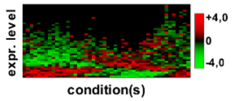	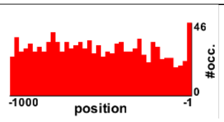	← 1.56e-03		
#20	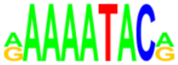	NA	1118	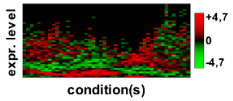	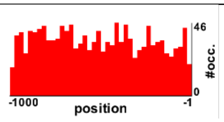	← 6.47e-05		
#21	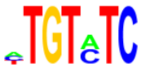	NA	1052	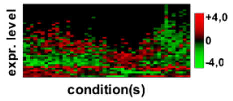	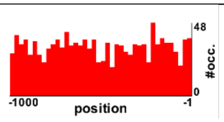			GO:0051809<br>passive evasion of immune response of...<br>P ≤1.53e-02
#22	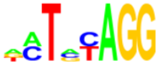	NA	991	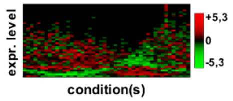	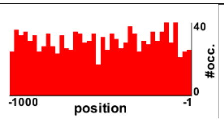			
#23	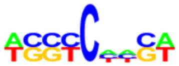	NA	983	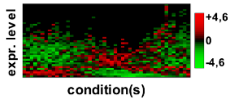	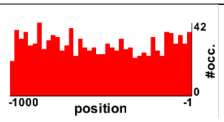			
