# Supplementary material for: Computational discovery of regulatory elements in a continuous expression space
Source: Genome Biol. 2012 Nov 27;13(11):R109. doi: 10.1186/gb-2012-13-11-r109 (PMC4053739; doi:10.1186/gb-2012-13-11-r109)
Supplement: Additional file 12 — Results of MatrixREDUCE on P. falciparum upstream regions with the Bozdech et al. dataset (erythrocytic cycle). The set of motifs inferred by MatrixREDUCE on the upstream regions of P. falciparum genes using the Bozdech et al. dataset [11]. See the description of Additional file 2 for table column definitions. [file gb-2012-13-11-r109-S12.PDF]

MatrixREDUCE on P.falciparum intraerythrocytic cycle (Bozdech et al.)								
id	logo	score	#genes	expression	distances	strand	match	GO terms
#1	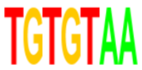	NA	904	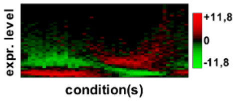	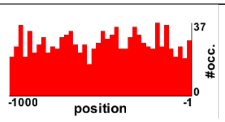	→ 1.26e-03	PFD0985w_D2 P $\leq$4.60e-04	
#2	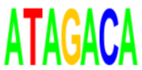	NA	328	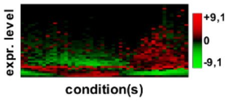	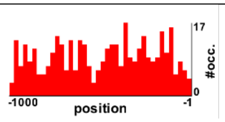			
#3	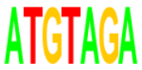	NA	641	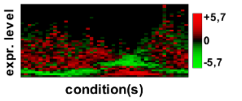	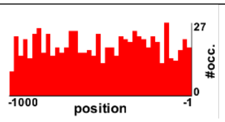	→ 3.80e-03	PFL1900w_D1 P $\leq$3.35e-02	
#4	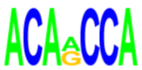	NA	212	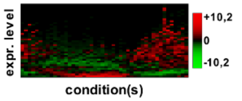	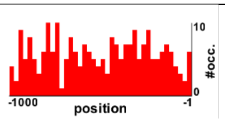			GO:0045177 apical part of cell P $\leq$6.63e-02
#5	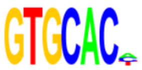	NA	133	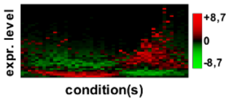	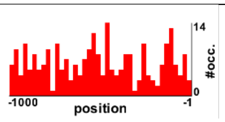		PF10_0075_D3 P $\leq$4.60e-04	GO:0005856 cytoskeleton P $\leq$2.39e-02
#6	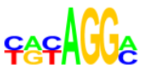	NA	1098	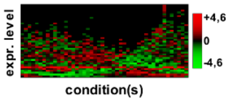	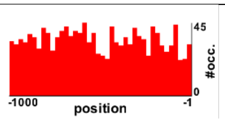			
#7	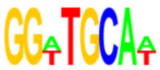	NA	60	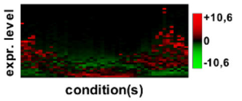	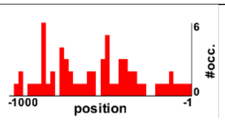			
#8	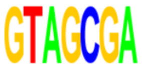	NA	11	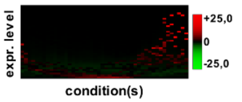	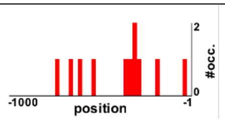			

#9	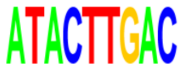ATACTTGAC	NA	5	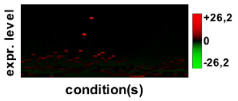expr. level<br>condition(s)<br>+26,2<br>0<br>-26,2	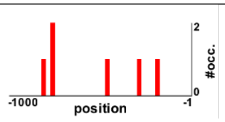#occ.<br>2<br>0<br>-1000<br>position<br>-1			
